# De Novo Transcriptome Analysis of *R. nigrum* cv. Aldoniai in Response to Blackcurrant Reversion Virus Infection

**DOI:** 10.3390/ijms23179560

**Published:** 2022-08-24

**Authors:** Ingrida Mažeikienė, Ana Dovilė Juškytė, Vidmantas Bendokas, Vidmantas Stanys

**Affiliations:** Lithuanian Research Centre for Agriculture and Forestry, Institute of Horticulture, Kaunas str. 30, 54333 Babtai, Lithuania

**Keywords:** BRV infection, biotic stress, blackcurrant, defense response, RNA-Seq, de novo transcriptome

## Abstract

The most damaging pathogen in blackcurrant plantations is mite-transmitted blackcurrant reversion virus (BRV). Some *Ribes* species have an encoded genetic resistance to BRV. We performed RNA sequencing analysis of BRV-resistant blackcurrant cv. Aldoniai to evaluate the molecular mechanisms related to the BRV infection response. The RNA of virus-inoculated and mock-inoculated microshoots was sequenced, and the transcriptional changes at 2- and 4-days post inoculation (dpi) were analyzed. The accumulation and expression of BRV RNA1 were detected in infected plants. In total, 159,701 transcripts were obtained and 30.7% were unigenes, annotated in 7 databases. More than 25,000 differentially expressed genes (DEGs) according to FPKM were upregulated or downregulated. We observed 221 and 850 DEGs at 2 and 4 dpi, respectively, in BRV-infected microshoots related to the stress response. The proportion of upregulated DEGs at 4 dpi was about 3.5 times higher than at 2 dpi. Pathways of the virus defense response were activated, and key candidate genes were identified. The phenylpropanoid and the cutin, suberine, and wax biosynthesis pathways were activated in infected plants. Our comparative de novo analysis of the *R. nigrum* transcriptome provides clues not only for understanding the molecular BRV resistance mechanisms but also for breeding BRV-tolerant genotypes.

## 1. Introduction

Several viruses can cause diseases in *Ribes* spp. Plant infections by viruses result in a range of symptoms, damage host plants, and adversely affect the economic value of blackcurrant plantations [1]. Blackcurrant reversion virus (BRV) is specific for currants and is the most damaging virus in *Ribes* spp. plantations worldwide. It is transmitted by the biological vector *Cecidophyopsis* spp. (Acari: *Eriophyidae*) in a persistent manner and mechanically through agrotechnical means and grafting. Atypical to other nepoviruses, BRV is not transmitted through seeds or pollen [2]. This viruscauses blackcurrant reversion disease (BRD) in European (E) or Russian (R) forms with several morphological distortions; the worst case is complete yield loss due to plants’ infertility [1,3,4].

BRV transmission that uses mechanical inoculation with sap or purified virions is complicated. The new method of transmission of BRV from BRV-positive *Ribes* to BRV-negative *Ribes* in vitro through root soaking in the inoculum was created earlier by [5].

BRV is a small (approximately 29 nm in diameter) icosahedral *Nepovirus* [6]. BRD etiology has been studied very sparsely; however, the positive-sense single-stranded RNAs of BRV have been characterized [7,8,9]. BRV is bipartite and has three isometric virion components: M with RNA1 (7711 bp), B with RNA2 (4600 bp), and T with satRNA (1432 bp), with all of them containing a single open reading frame (ORF). RNA2 encodes 55 or 54 kDa coat protein (CP). BRV particles with 54 kDa CP are suitable for mechanical inoculation through leaf abrasion [6].

*Ribes nigrum* cv. Aldoniai is a new cultivar that was created at the LAMMC in 2017. Aldoniai is distinguished as a high-yielding, self-pollinating, gall-mite-resistant and BRV-resistant cultivar [10]. The cultivar combines various blackcurrant genotypes and is a descendant of *R. dikuscha* Fisch., *R. nigrum* spp. *sibiricum* Pavl., and *R. nigrum* spp. *europaeum* Pavl. Resistance to BRV in cv. Aldoniai was inherited from *R. dikuscha*. Additionally, this cultivar has pyramidic resistance to gall mite and BRV according to previous studies involving molecular markers [11].

To survive, plants have evolved multiple sophisticated and complex regulatory mechanisms to defend themselves against virus infection, including gene silencing pathways, hormone-mediated signaling pathways, and metabolism regulation [12]. Studies of the molecular basis of BRV resistance have focused mainly on qualitative resistance and some molecular markers related to resistance to BRV were suggested [13,14]. Although resistance to BRV in *Ribes* spp. appears to be a quantitative trait controlled by a dominant gene [15,16], the genetic control of resistance is complex and remains unknown.

Hence, it is necessary to attain a comprehensive understanding of the molecular mechanisms of resistance to BRV in *Ribes* for novel blackcurrant breeding programs and acceleration of the selection process. Recently, RNA-sequencing (RNA-Seq) technology and digital gene expression analysis have provided new and rapid approaches for detecting differences in the transcriptomes of non-model plants [17,18,19]. Global investigation of gene expression during BRV infection will help to elucidate the mechanisms of BRV resistance in *Ribes* plants. RNA-seq, de novo transcriptome assembly, and flavonoid gene analysis of blackcurrant berries were studied [18]. Transcriptome analysis of blackcurrant’s response to biotic stress has not been performed, and data analysis of genetic studies of the single genus *Ribes* from the order Saxifragales is complicated due to a lack of a possibility of comparison to genetic data of other reference genera in Eudicots (*Rosids* and *Asterids*) [18,20].

In this study, we used next-generation sequencing approaches on the BRV-resistant blackcurrant cultivar to analyze the responses to BRV infection at the transcriptomic level. We investigated the differences in gene expression between virus-infected and mock-inoculated samples at different time points following BRV infection (at 2 and 4 days after infection). Our results showed that some plant defense and virus resistance genes were differentially expressed between the infected plants and control. Our study provides insight into the molecular mechanism of blackcurrant’s response/resistance to BRV infection and advances our understanding of plant–virus interactions. 

## 2. Results

### 2.1. Quality Control of BioProject Data

Twelve cDNA libraries of four experiment treatments C_2, V_2, C_4, and V_4 were generated for RNA-Seq of *R. nigrum* cv. Aldoniai to study genes and pathways related to resistance to BRV. More than 305 million high-quality clean reads were produced from microshoot RNA and were used for performing de novo transcriptome assembly of *R. nigrum*. The raw data obtained by NGS in this study is available from the NCBI database (accession PRJNA797914 of BioProject). In total, 92.9 Gbases of raw reads of SRA data per BioProjec were generated (Table 1). Clean reads accounted for 9.85%, comprising 6.3 to 8.9 Gbases among samples with a Q20 percentage of 97.02–97.55% and a GC percentage of 42.61–43.60%.

**Figure 1:**
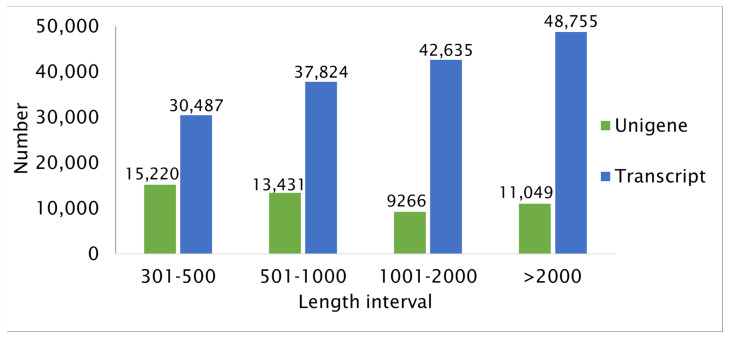
Length distribution of the assembled transcripts and unigenes from the transcriptome of *R. nigrum* cv. Aldoniai.

More than 159,701 transcripts were assembled and a total of 48,966 non-redundant unigenes were generated. A total of 15,220 unigenes (31.08%) had a sequence length of up to 500 bp and 13,431 unigenes (27.43%) had a length of 501–1000 bp. In addition, 9266 unigenes (18.92%) had a length of 1001–2000 bp, and 11,049 unigenes (22.56%) had a sequence longer than 2000 bp. As a result, the number of transcripts assembled in this project increased as the sequence length increased; however, the percent of the assembled number of unigenes decreased despite the increasing number of transcripts for a given length in bp (Figure 1).

### 2.2. Annotation of Unigenes in R. nigrum Transcriptome

**Figure 2:**
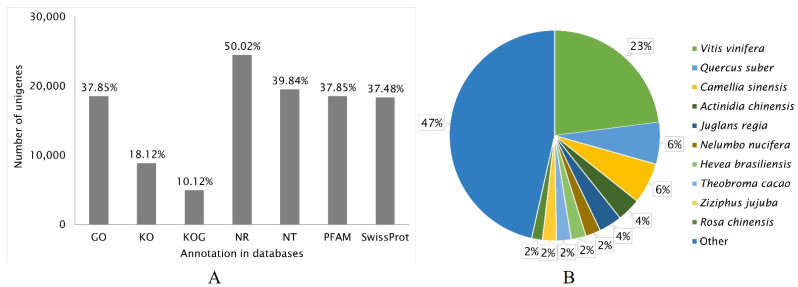
Number of unigenes of blackcurrant cv. Aldoniai annotated into different databases (**A**) and the identity (round value) of plant species to *R. nigrum* gene annotation according to the NR database (**B**).

All assembled unigenes (48,966) obtained by sequencing of the *R. nigrum* transcriptome were annotated into seven databases: GO, KO, KOG, NR, NT, PFAM, and SwissProt (Figure 2A). The largest number of unigenes 24,497 (50.02%) in the de novo assembled genome were identified and functionally annotated by the NR database. The annotation data according to the databases GO, NT, PFAM, and SwissProt were similar, and 37.85%, 39.84%, 37.85%, and 37.48% of the unigenes were identified, respectively. Finally, the least number of functionally annotated genes were identified according to the data of KO and KOG, with 18.12% and 10.12%, respectively.

The highest matching percent of 23.0% in the *R. nigrum* unigene sequences was with the genes of *Vitis vinifera* according to the NR database (Figure 2B). The assembled unigenes showed a 6.4% phylogenetic identity with the genes of *Quercus suber*, and 6.3% with the genes of *Camellia sinensis*. We established a lower genetic identity between blackcurrant and *Actinidia chinensis* and *Juglans regia*, which was 3.6% in both cases. About half (47.0%) of the assembled unigenes did not match the protein sequences of other plant species. These unigenes, with lengths of 150–450 bp, were specific and unique to *R. nigrum* and have not been identified yet. Further, their protein sequences do not match with proteins from other plant species.

**Figure 3:**
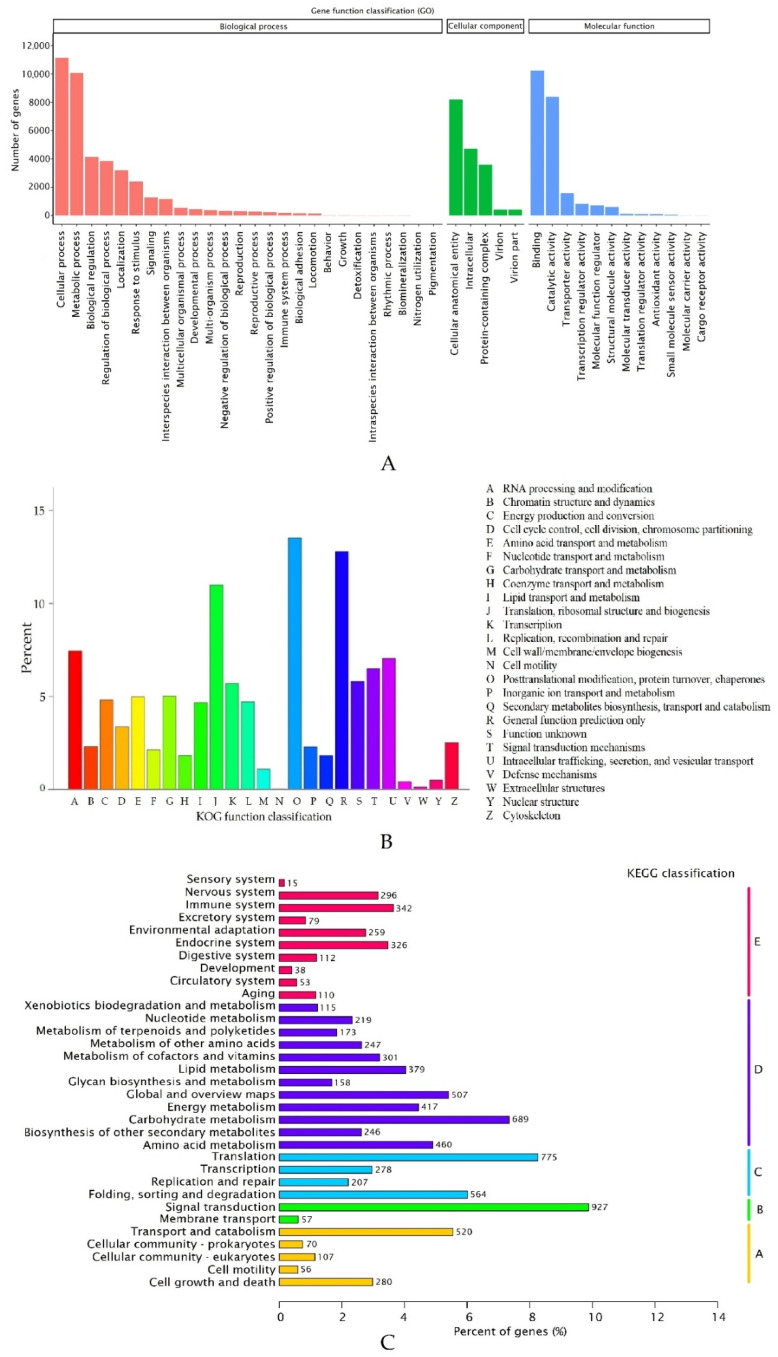
Characteristics of blackcurrant unigenes based on Gene Ontology (GO) categorization (**A**), Clusters of Orthologous Group (KOG) classification (**B**), and Kyoto Encyclopedia of Genes and Genomes (KEGG) pathways classification (**C**). The *x*-axis is the names of the 25 KOG group, and the *y*-axis is the percentage of genes annotated under this group of the total annotated genes. The *y*-axis is the names of the KEGG pathways, and the *x*-axis is the number of genes annotated in the pathway and the ratio between the number in this pathway and the total number of annotated genes. The KEGG metabolic pathway genes are divided into 5 branches: A: Cellular Processes, B: Environmental Information Processing, C: Genetic Information Processing, D: Metabolism, E: Organismal Systems.

The functions of *R. nigrum* unigenes were annotated using GO, KEGG, and KOG analysis data (Figure 3). Using GO analysis, 80,470 unigenes were grouped into 3 functional categories (biological process, cellular component, and molecular function) and assigned to 43 functional groups (Figure 3A). In total, 40,335 unigenes identified in the *R. nigrum* microshoot transcriptome were related to genes involved in 26 various biological processes. In total, 11,133 and 10,081 unigenes were involved in cellular and metabolic processes, respectively. In total, 4135 and 3852 unigenes were involved in biological regulation and regulation of biological process. In total, 3199 unigenes were involved in localization and 2395 unigenes were involved in response to stimulus. Fewer of the identified unigenes were involved in signaling (1279) and interspecies interaction between organisms (1162), 40 of which were associated with the response to virus. We also identified unigenes related to the multicellular organism (541), development (444), multi-organism (387), negative regulation (327), reproduction (295), and other processes, as shown in Figure 3A. In total, 17,358 unigenes were divided into 5 subcategories in the cellular component category. In total, 22,777 unigenes were involved in molecular function and they were distributed into 12 subcategories, in which unigenes from the binding (10,243) and catalytic activity (8384) subcategories dominated.

The functions of *R. nigrum* unigenes were predicted and classified by searching the KOG database (Figure 3B). In total, 4957 unigenes were classified into 25 groups composed of ancestral proteins. *R. nigrum* unigenes were assigned in all groups via KOG. Three dominant groups of unigenes in *R. nigrum* emerged according to the KOG classification data. Post-translational modification, protein turnover, and chaperones group (O) consisted of 671 unigenes (13.54%); general function prediction only group (R) included 634 unigenes (12.79%); and the translation, ribosomal structure, and biogenesis group (J) included 545 unigenes (10.99%). However, the smallest groups were extracellular structures (W) and cell motility (N), with seven and two unigenes, respectively.

All annotated unigenes (9382) were classified into five major metabolic pathways: 1033 in cell process (A), 984 in environmental information processing (B), 1824 in genetic information processing (C), 3911 in metabolism (D), and 1630 in organismal systems (E) via the KEGG database (Figure 3C). The five metabolic pathways were further divided into 33 subcategories. In total, 41.69% of the unigenes were annotated to the metabolism pathways. A maximum number of unigenes of 927 were related to signal transduction metabolism, 775 unigenes were related to translation, and 689 unigenes were related to carbohydrate metabolism.

### 2.3. Differentially Expressed Genes (DEGs) to Virus Defense Response in R. nigrum cv. Aldoniai Microshoots

**Figure 4:**
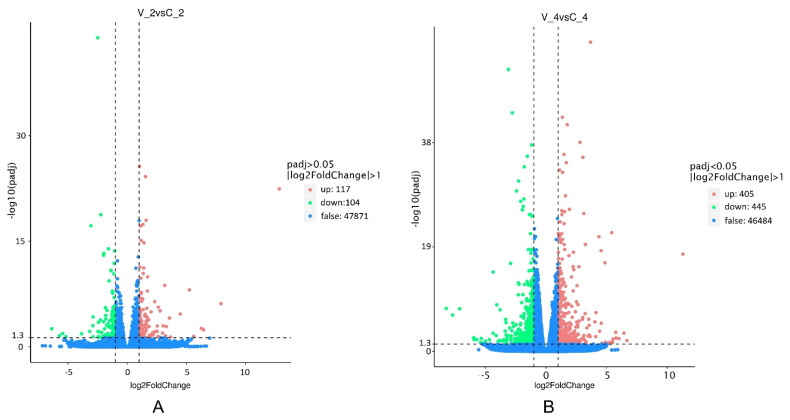
The number of differentially expressed genes (DEGs) in response to BRV infection at 2 (**A**) and 4 dpi (**B**) in *R. nigrum* cv. Aldoniai. The threshold is normally set as: [log2 (Fold Change)] > 1 and q-value < 0.005. The *x*-axis shows the fold change in the gene expression between different samples, and the *y*-axis shows the statistical significance of the differences. Statistically significant differences are represented by red or green dots.

Using the de novo assembled transcriptome as a reference, the genes expressed in the virus-inoculated and mock-inoculated (control) groups after 2 and 4 dpi were identified. In total, 48,092 genes after 2 dpi and 47,334 genes after 4 dpi were evaluated (Figure 4). The padj ≤ 0.05 and log2 ratio ≥ 1 were used to identify DEGs in the virus-inoculated samples in comparison to control plants. DEGs increased in virus-infected plants by approximately 4 times during the post-inoculation period from 2 to 4 days. In total, at 2 dpi, 221 DEGs were identified, of which 104 were downregulated and 117 were upregulated in response to BRV infection (Figure 4A and Table S1). The fold change in gene expression varied between one and six. A significant increase in DEGs (850 genes) in virus-inoculated samples was determined at 4 dpi, with 445 genes being downregulated and 405 being upregulated (Figure 4B and Table S1). The expression level remained unchanged (false) for 99.54% (47,871) of the genes at 2 dpi and 98.20% (46,484) of the genes at 4 dpi.

Plants in this in vitro inoculation assay were infected with inoculum with three different BRV isolates [5]. The expression of viral polyprotein encoded by RNA1 (cluster-12591.29271) was detected in virus-inoculated plants. The log2FoldChange (log2FC) in the microshoots was 12.85 (q value 3.4704 × 10^−23^) at 2 dpi and 11.29 (q value 2.0349 × 10^−18^) at 4 dpi (Figure 4 and Table S1). Three polyproteins of BRV with genetic diversity in strand RNA2 were identified in the transcriptomes of infected blackcurrant in Trinity data (Figure S1), but clean reads were not annotated according to databases, and the expression of virus RNA2 in microshoots of blackcurrant was not observed.

**Figure 5:**
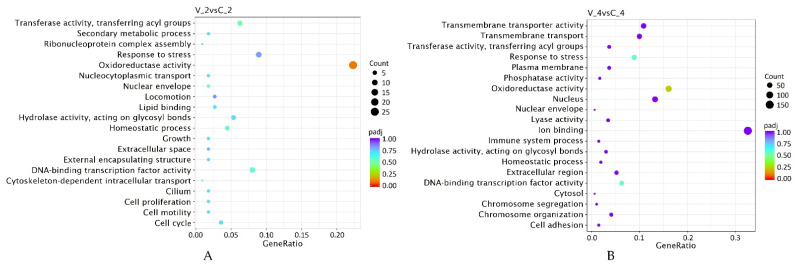
Enriched gene pathways identified according to GO involving differentially expressed genes (DEGs) following biotic stress (virus) inoculation at 2 (**A**) and 4 dpi (**B**) in *R. nigrum* cv. Aldoniai.

The enriched gene pathways identified by GO (functional categories grouping) are presented in Figure 5. Twenty functional pathways with reliable expression of specific genes were identified in each post-inoculation period. However, the structure of enriched gene pathways was partially different between the studied periods. Specifically, for the activated pathways, the DEG characteristics are presented in the Supplementary Materials (Table S2). Statistically significantly upregulated DEGs (gene ratio 19:65) at 2 dpi were identified in the molecular function oxidoreductase activity (Figure 5A). Meanwhile, at 4 dpi, the oxidoreductase activity function genes were downregulated at a ratio of 54:256, and the biological process response to stress was upregulated at a gene ratio of 26:204 (Figure 5B).

**Figure 6:**
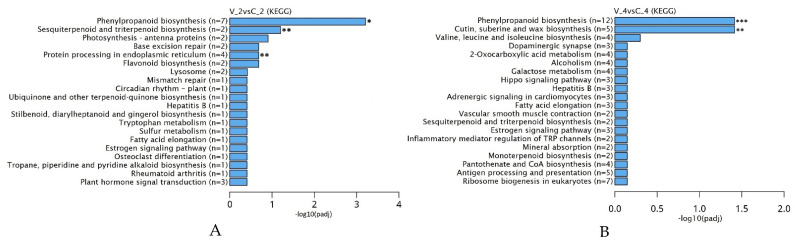
(**A**,**B**) Kyoto Encyclopedia of Genes and Genomes (KEGG) pathway enrichment analysis of up- and downregulated genes (n = x). All pathways were enriched using the criterion *p* < 0.05. *—significant upregulation, **—significant downregulation, ***—significant up- and downregulation of the pathways.

The KEGG enrichment scattered plot shows the DEGs enrichment analysis results in twenty KEGG pathways. The significant interactions of 13 genes at 2 dpi and 17 genes at 4 dpi show genes were involved in certain biological functions via KEGG analysis (Figure 6 and Table S3). Two days after BRV inoculation, unigenes in the phenylpropanoid biosynthesis were upregulated (ratio 7:23). Meanwhile, unigenes in the protein processing in the endoplasmic reticulum pathway (gene ratio 4:13) and sesquiterpenoid and triterpenoid biosynthesis (gene ratio 2:13) showed significant reductions in the expression levels. Although the plant hormone signal transduction pathway (gene ratio 3:13) had separate reliably expressed genes, the pathway itself was not statistically reliable. Four days after BRV infection, five genes with upregulation in phenylpropanoid biosynthesis were appointed; however, there was also negative expression of seven genes in this pathway. Negative signaling in the cutin, suberine, and wax biosynthesis (gene ratio 5:103) was observed in this period also.

## 3. Discussion

### 3.1. Transcriptome of R. nigrum Microshoots

We conducted de novo assembly of transcriptomes of BRV-inoculated and mock-inoculated microshoots in BRV-resistant blackcurrant cv. Aldoniai. *R. nigrum* belongs to the Saxifragales order and *Grossulariaceae* family; molecular-genetic studies of plants from this family are very sparce. The nutritional and medicinal values of currant fruits are indisputable, and have stimulated research on the synthesis of secondary metabolites in fruits. Several genetic studies related to fruit ripening and secondary metabolite synthesis have been performed based on RNA transcripts [18,21,22]. Our main attention in this study was concentrated on the ecological aspect of plant cultivation since BRV pathogen and BRD cannot be treated and cause great economic losses. Consequently, the development of virus-resistant plants is a relevant task for breeders. Although studies of the genetically determined disease resistance of the *Ribes* genus have been carried out for a long time [3,15], a system of molecular marker usage was offered to breeders [11,14,23]. The resistance or tolerance of individual *Ribes* spp. genotypes of BRV is determined genetically, and markers are created, but the host plant–pathogen interaction at the genetic level has not been studied. De novo transcriptome assembly using NGS was performed for the first time in BRV-infected plants according to the methodology under in vitro conditions created earlier [5]; this method opened possibilities to study specific and precise host–virus interactions in *Ribes* spp. 

Aldoniai is the new BRV-resistant Lithuanian cultivar [10] with the genealogy of interspecific hybrids. This cultivar was used as a model because the virus may be observed in plant material up to 6–8 days after inoculation and significant expression changes in *pathogenesis-related 1* (*PR1*) were detected at 2 and 4 dpi as stated in previous studies. This gene was used as a molecular marker to indicate blackcurrant’s defense response to BRV infection [5,24]. Therefore, the transcriptomes of cv. Aldoniai microshoots (mock- and BRV-inoculated) at 2 and 4 dpi were analyzed (Table 1). Raw reads from 21,330,139 to 30,235,731 were received in the mRNA of microshoots while 168,239,621 raw reads in ripe fruits of blackcurrants cv. Ben Hope were counted [18]. The *Ribes* spp. genome lacked an available reference genome sequence; therefore, de novo assembly of the Illumina reads was carried out for cv. Aldoniai using Trinity software and 159,701 transcripts were assembled (Figure 1). This number of transcripts is higher than in cv. Ben Hope fruits (145,906 transcripts) but lower in comparison to *R. nigrum* var. *sibiricum* (186,129 transcripts) [18]. The Siberian subspecies are one of the ancestors of the cv. Aldoniai. In total, 48,966 non-redundant unigenes were generated in our study and only half of them were annotated in the NR database. Among the homologous sequences of other plant species, the matching degree with *V. vinifera* was the highest (Figure 2). Similar results were obtained while studying fruit transcriptomes and performing phylogenetic analysis of plants [18]. According to the time of plant species divergence and comparative analysis of plant transcriptomes, *Ribes* spp. (Saxifragales) diverged about 117 million years ago from the Vitales and most genes related to biological processes and molecular functions etc. are characterized by high genetic diversity [18,25]. Therefore, they are not identified in the NCBI Blast database at the nucleotide and amino acid levels. Conservative genes are better recognized in the biological cellular and metabolic processes or molecular functions binding and catalytic activity (Figure 3).

### 3.2. Virus Defense Response to BRV in R. nigrum

In our study, expression differences were observed in 47,871 genes. DEGs related to virus infection with a significant value of log2FC increased at 4 dpi in comparison to 2 dpi in BRV-infected blackcurrants (Figure 4).

BRV infection was approved by the presence of virus RNA1 and RNA2 in the transcriptome of inoculated samples (Figure S1). According to the data of a previous study [5], inoculum with three different BRV isolates was used in our experiment. RNA2 strand is responsible for translation of three proteins: X, movement protein (MP), and coat protein (CP). However, the expression of polyprotein from RNA2 did not occur in virus-inoculated samples at 2 or 4 dpi. An analysis of DEGs showed that polyprotein from RNA1 of BRV were strongly upregulated in virus-infected *R. nigrum* microshoots. 

RNA1 encodes five proteins: N-terminus protease co-factor, followed by nucleotide-binding protein or helicase, putative VPg, cysteine protease (CysPro), and RNA-dependent RNA polymerase [9]. Different genes of the virus are recognized as plant virus avirulence (*Avr*) factors in the viral Avr-R system [26]. Only polyprotein of BRV RNA1 showed significant expression (Figure 4 and Table S1) (log2FC 12.85 and 11.29 at 2 and 4 dpi, respectively) and could be recognized as an *Avr* gene in blackcurrant’s plant–pathogen interaction pathway. We assume that the genes of BRV RNA1 can activate the chemical defense in *R. nigrum*. A different genetic response of the blackcurrants was observed due to BRV-induced stress at 2 and 4 dpi (Figure 5). A change in the reduction–oxidation status in plant cells is one of the earliest responses to biotic stress. The production of reactive oxygen species (ROS) and nitric oxide (NO) and their compounds synergistically activates the hypersensitive response (HR) in model plants [27]. In our case, genes from the oxidoreductase activity pathway (GO:0016491) were the first upregulated genes in the virus-infected samples of *R. nigrum* at 2 dpi (Figure 5, Table S2). At 4 dpi, oxidoreductase activity genes were suppressed; however, at this moment, the biological process response to stress (GO:0006950) was significantly upregulated in the microshoots. We propose that at 4 dpi, the microshoots of cv. Aldoniai reacted to artificially induced stress.

Some plant compound elicitors (benzoic acid, chitosan, salicylic acid, etc.) that activate the defense in plants were studied [26]. Synthesis of the signal-mediating phytohormones: salicylic acid (SA) (at 2 dpi), jasmonic acid (JA), and ethylene (at 4 dpi) was observed in the plant hormone signal transduction pathway (ko:04075) in BRV-infected plants (Figures S2 and S3). These phytohormones stimulate plant responses to different biotic and abiotic stresses, leading to activation of systemic acquired resistance (SAR) and induced systemic resistance (ISR) [28,29]. It was established that both SAR and ISR were activated in BRV-infected microshoots.

Various biosynthetic pathways can be activated in plants depending on the elicitor compound stimulation [30]. Several of the upregulated genes from the phenolic compound biosynthesis pathway identified in BRV-infected microshoots were related to the activation of different defense-related enzymes (peroxidases) (Table S2). The phenylpropanoid biosynthesis pathway (ko:00940) was significantly upregulated at both 2 and 4 dpi in our experiment (Figure 6). The synthesis of some compounds, including coumarine, cinnamaldehyde, caffeoyl quinic acid, and lignin, was increased at 2 dpi (Figure S4). The synthesis of ferulic acid, sinapis acid, caffeoyl-aldehyde, caffeoyl-alcohol, 5-Hydroxy-coniferaldehyde, and 5-Hydroxy-coniferyl alcohol was also increased at 4 dpi while the synthesis of cinnamic acid was suppressed. Some lignin synthesis genes in *R. nigrum*-infected samples were up- and downregulated at 4 dpi (Figure S5). Consequently, the responses to virus infection in blackcurrants appeared through the synthesis of phenylpropanoids, which also play important roles in the response to biotic stress in other plants [31].

Cuticular wax biosynthesis and its roles in plant disease resistance are well established [32]. Negative signaling of the genes in the cutin, suberine, and wax biosynthesis pathway (ko:00073) in virus-inoculated plants was observed at 4 dpi (Figure S6). Suppression of the synthesis of fatty acids in the plant defense system must be related to the production of signaling molecules JA (oxylipin) for defense regulation, with remodeling of the membrane lipid composition and defense signaling [33]. Long-chain fatty acid synthesis suppression has a negative effect on the biosynthesis of the plant cuticle and the generation of bioactive molecules, including sphingolipids. Therefore, BRV infection leads to the suppression of the ko:00073 pathway, thereby reducing the plant’s resistance, and thus facilitating the entry of other pathogens such as bacteria and fungi into the plant cells.

In this study, we reported a transcriptome analysis that includes data on blackcurrant’s response to an economically important and very harmful pathogen: BRV. Such data is necessary for gene identification and functional analysis to help improve gene resources. These resources will enable investigations of the stress response, defense response, and metabolic processes in plants beyond the *R. nigrum*–BRV interaction pathways analyzed in this study. Our results clarified that blackcurrant cv. Aldoniai (resistant to BRV interspecific hybrid) has a complex of genetically determined defense mechanisms aimed at BRV infection, which were significantly activated 2 and 4 days after viral infection.

## 4. Materials and Methods

### 4.1. RNA Isolation, cDNA Library Preparation, and Sequencing

Plants of cv. Aldoniai were inoculated with BRV under in vitro conditions at the Department of Orchard Plant Genetics and Biotechnology of the Institute of Horticulture, LAMMC according to the methodology described by [5]. Control samples of mock- (C) and virus-inoculated (V) microshoots were collected at 2- and 4-days post inoculation (dpi) (sample library ID C_2 and V_2, C_4 and V_4) (Table 1). Three individual microshoots from each treatment were collected to provide biological replicates. Samples were immediately frozen in liquid nitrogen and stored until RNA extraction with a GeneJET Plant RNA Purification Mini Kit (Thermo Scientific, Vilnius, Lithuania) according to the manufacturer’s instructions. The concentration and quality of the RNA were measured with an Implen GmbH spectrophotometer (Implen, Munich, Germany).

### 4.2. De Novo Transcriptome Analysis

Qualified RNA, with 4 μg per sample, was sent to Novogene (Cambridge, UK) for mRNA library preparation (poly-A enrichment). The next-generation sequencing (NGS) was conducted on an Illumina 6000 NovaSeq PE150 (6 Gb raw data per sample) platform. In total, the data of 36 replicates (3 biological replicates per treatment and 3 techniques) were used in the de novo transcriptome analysis of *R. nigrum* cv. Aldoniai.

### 4.3. Functional Annotation of Unigenes

Low-quality raw reads were filtered as follows: reads containing adaptors were removed, N > 10% and with Q score of over 50% bases below 5 (Table 1). The de novo transcriptome (absence of a reference genome) was reconstructed using Trinity [34]. Assembled transcripts were clustered with the command-line software program Corset [35], and redundancy was removed. The longest transcripts of each cluster were selected as unigenes.

### 4.4. Gene Functional Annotation

Functional annotation of all assembled unigenes was performed using seven public databases: Non-Redundant Protein Sequence Database (NR), Nucleotide Sequence Database (NT), Gene Ontology (GO), Swiss-Prot, Kyoto Encyclopedia of Genes and Genomes (KEGG), Pfam, and Clusters of Orthologous Groups for Eukaryotic Complete Genomes (KOG). The genes successfully annotated in GO were grouped into three main domains: Biological Process (BP), Cellular Component (CC), and Molecular Function (MF). Genes annotated in KOG were divided into functional groups. The genes successfully annotated in KEGG were classified according to the KEGG pathway they joined in.

### 4.5. Gene Expression Analysis

To compare the gene expression levels under different conditions, the FPKM (Fragments Per Kilobase of transcript per Million mapped reads) values were used. For biological replicates, the final FPKM was the mean value. The FPKM values were transformed to log2FoldChange (log2FC); log2FC ≥ 1 was considered to have significant expression.

## Figures and Tables

**Table 1:** Summary data of quality control (QC) of reads of the genome *R. nigrum* cv. Aldoniai.

Sample Library ID	Raw Reads	Raw Bases, G	Clean Reads	Clean Bases, G	Error Rate, %	Q20	GC_pct, %
C_2_1	28,086,156	8.4	27,637,344	8.3	0.03	97.45	43.53
C_2_2	24,977,381	7.5	24,594,501	7.4	0.03	97.51	43.32
C_2_3	24,061,978	7.2	23,627,353	7.1	0.03	97.55	43.36
V_2_1	27,826,308	8.3	27,343,527	8.2	0.03	97.52	43.57
V_2_2	24,844,092	7.5	24,476,571	7.3	0.03	97.28	43.49
V_2_3	26,612,831	8.0	26,276,865	7.9	0.03	97.41	43.47
C_4_1	23,144,738	6.9	22,864,209	6.9	0.03	97.50	43.60
C_4_2	21,330,139	6.4	21,028,990	6.3	0.03	97.18	43.27
C_4_3	30,235,731	9.1	29,812,238	8.9	0.03	97.25	43.36
V_4_1	23,810,909	7.1	23,507,940	7.1	0.03	97.21	42.93
V_4_2	28,857,614	8.7	28,387,016	8.5	0.03	97.58	42.61
V_4_3	25,987,937	7.8	25,614,722	7.7	0.03	97.02	42.80

Error rate (%)—base error rate of whole sequencing; Q20—the percentage of bases whose Q Phred values are greater than 20; GC pct (%)—the percentage of G and C base numbers of the total bases.

## Data Availability

This study did not report any data.

## Supplementary Materials

The supporting information can be downloaded at: https://www.mdpi.com/article/10.3390/ijms23179560/s1.

## References

[1] Šutic D.D., Ford R.E., Tošic M.T., Šutic D.D., Ford R.E., Tošic M.T. (1999). Virus diseases of small fruits. Handbook of Plant Virus Diseases.

[2] Dolan A., MacFarlane S.A., McGavin W.J., Brennan R.M., McNicol J.W. (2011). Blackcurrant reversion virus: Validation of an improved diagnostic test, accelerating testing in breeding and certification of blackcurrants. J. Berry Res..

[3] Adams A.N., Thresh J.M., Converse R.H. (1987). Reversion of black currant. Virus Diseases of Small Fruits.

[4] Susi P. (2004). Black currant reversion virus, a mite-transmitted nepovirus. Mol. Plant Pathol..

[5] Juškytė A.D., Mažeikienė I., Stanys V. (2022). An effective method of *Ribes* spp. inoculation with blackcurrant reversion virus under in vitro conditions. Plants.

[6] Seitsonen J.J.T., Susi P., Lemmetty A., Butcher S.J. (2008). Structure of the mite-transmitted *Blackcurrant reversion nepovirus* using electron cryo-microscopy. Virology.

[7] Latvala-Kilby S., Lehto K. (1999). The complete nucleotide sequence of RNA2 of blackcurrant reversion nepovirus. Virus Res..

[8] Pacot-Hiriart C., Latvala-Kilby S., Lehto K. (2001). Nucleotide sequence of black currant reversion associated nepovirus RNA1. Virus Res..

[9] Moročko-Bičevska I., Stalažs A., Lācis G., Laugale V., Baļķe I., Zuļģe N., Strautiņa S. (2021). *Cecidophyopsis* mites and blackcurrant reversion virus on *Ribes* hosts: Current scientific progress and knowledge gaps. Ann. Appl. Biol..

[10] Mažeikienė I., Stanys V., Juškytė A.D., Sasnauskas A., Šikšnianas T. (2017). Juodojo serbento veislės ‘Aldoniai’ ir ‘Didikai’. Sodininkystė Daržininkystė.

[11] Mazeikiene I., Juskyte A.D., Stanys V. (2019). Application of marker-assisted selection for resistance to gall mite and Blackcurrant reversion virus in *Ribes* genus. Zemdirbyste.

[12] Ding S.W. (2010). RNA-based antiviral immunity. Nat. Rev. Immunol..

[13] Brennan R., Jorgensen L., Hackett C., Woodhead M., Gordon S., Russell J. (2008). The development of a genetic linkage map of blackcurrant (*Ribes nigrum* L.) and the identification of regions associated with key fruit quality and agronomic traits. Euphytica.

[14] Brennan R.M., Jorgensen L., Gordon S., Loades K., Hackett C., Russell J. (2009). The development of a PCR-based marker linked to resistance to the blackcurrant gall mite (*Cecidophyopsis ribis* Acari: *Eriophyidae*). Theor. Appl. Genet..

[15] Anderson M.M. (1971). Resistance to gall mite (*Phytoptus ribis* Nal.) in the Eucoreosma section of *Ribes*. Euphytica.

[16] Keep E., Knight V.H., Parker J.H. (1982). Progress in the integration of characters in gall mite resistant black currants. J. Hortic. Sci..

[17] Deng S., Ma J., Zhang L., Chen F., Sang Z., Jia Z., Ma L. (2019). De novo transcriptome sequencing and gene expression profiling of *Magnolia wufengensis* in response to cold stress. BMC Plant Biol..

[18] Thole V., Bassard J.E., Ramírez-González R., Trick M., Ghasemi Afshar B., Breitel D., Hill L., Foito A., Shepherd L., Freitag S. (2019). RNA-seq, de novo transcriptome assembly and flavonoid gene analysis in 13 wild and cultivated berry fruit species with high content of phenolics. BMC Genom..

[19] He D., Zhang J., Zhang X., He S., Xie D., Liu Y., Li C., Wang Z., Liu Y. (2020). Development of SSR markers in *Paeonia* based on de novo transcriptomic assemblies. PLoS ONE.

[20] Simpson M.G., Simpson M.G. (2019). Diversity and classification of flowering plants: Eudicots. Plant Systematics.

[21] Jarret D.A., Morris J., Cullen D.W., Gordon S.L., Verrall S.R., Milne L., Hedley P.E., Allwood J.W., Brennan R.M., Hancock R.D. (2018). A transcript and metabolite atlas of blackcurrant fruit development highlights hormonal regulation and reveals the role of key transcription factors. Front. Plant Sci..

[22] Starkevič P., Ražanskienė A., Starkevič U., Kazanavičiūtė V., Denkovskienė E., Bendokas V., Šikšnianas T., Rugienius R., Stanys V., Ražanskas R. (2020). Isolation and analysis of anthocyanin pathway genes from *Ribes* genus reveals MYB gene with potent anthocyanin-inducing capabilities. Plants.

[23] Mazeikiene I., Bendokas V., Stanys V., Siksnianas T. (2012). Molecular markers linked to resistance to the gall mite in blackcurrant. Plant Breed..

[24] Juškytė A.D., Mažeikienė I., Stanys V. (2022). Putative genes of pathogenesis-related proteins and coronatine-insensitive protein 1 in *Ribes* spp.. Plants.

[25] One Thousand Plant Transcriptomes Initiative (2019). One thousand plant transcriptomes and the phylogenomics of green plants. Nature.

[26] Huang C. (2021). From player to pawn: Viral avirulence factors involved in plant immunity. Viruses.

[27] Matika D.E.F., Loake G.J. (2014). Redox regulation in plant immune function. Antioxid. Redox Signal..

[28] Alazem M., Lin N.S. (2015). Roles of plant hormones in the regulation of host-virus interactions. Mol. Plant Pathol..

[29] Kamle M., Borah R., Bora H., Jaiswal A.K., Singh R.K., Kumar P., Hesham A.L., Upadhyay R., Sharma G., Manoharachary C., Gupta V. (2020). Systemic acquired resistance (SAR) and induced systemic resistance (ISR): Role and mechanism of action against phytopathogens. Fungal Biotechnology and Bioengineering.

[30] Thakur M., Sohal B.S. (2013). Role of elicitors in inducing resistance in plants against pathogen infection: A review. Int. Sch. Res. Not..

[31] Vogt T. (2010). Phenylpropanoid biosynthesis. Mol. Plant.

[32] Wang X., Kong L., Zhi P., Chang C. (2020). Update on cuticular wax biosynthesis and its roles in plant disease resistance. Int. J. Mol. Sci..

[33] Raffaele S., Leger A., Roby D. (2009). Very long chain fatty acid and lipid signaling in the response of plants to pathogens. Plant Signal. Behav..

[34] Grabherr M.G., Haas B.J., Yassour M., Levin J.Z., Thompson D.A., Amit I., Adiconis X., Fan L., Raychowdhury R., Zeng Q. (2011). Full-length transcriptome assembly from RNA-Seq data without a reference genome. Nat. Biotechnol..

[35] Davidson N.M., Oshlack A. (2014). Corset: Enabling differential gene expression analysis for *de novo* assembled transcriptomes. Genome Biol..

