# Supplementary material for: De Novo Transcriptome Analysis of R. nigrum cv. Aldoniai in Response to Blackcurrant Reversion Virus Infection
Source: Int J Mol Sci. 2022 Aug 24;23(17):9560. doi: 10.3390/ijms23179560 (PMC9455767; doi:10.3390/ijms23179560)
Supplement: Supplementary file 1 [file ijms-23-09560-s001.zip › Supplements/Figure S1.pdf]

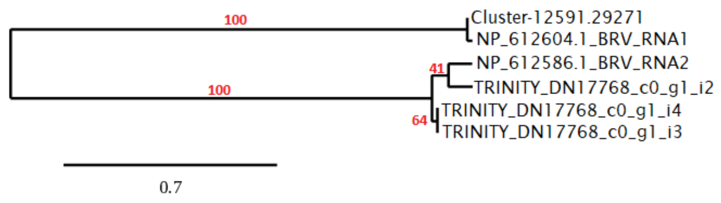

Figure S1: Phylogenetic tree of BRV RNAs at amino acid level and Trinity and Cluster sequences (shown in fasta format below) assembled in BRV-inoculated cv. Aldoniai. Accession numbers of sequences NP_612604.1 and NP_612586.1 according to NCBI database [7,8]. Phylogenetic analysis was performed using the maximum likelihood method implemented in the PhyML program (online version http://phylogeny.lirmm.fr/phylo_cgi/index.cgi); a bootstrap analysis with 100 replications was performed.

RNA1 sequence in transcriptome of BRV-inoculated cv. Aldoniai

Underlined parts of sequences are translated to amino acids

```
>Cluster-12591.29271
CTTTTGCCTCCTCTTTCTCCTTGTCTTCTTTTATTGATTTTACAATGGTGAAAATTGGTTCCGTCACCCTCGCTCCC
AAGCAACTGGTTCTTGAGAACAAATATATTGCTGCGTGCCTTTCTAGGCCAGCAAATTTTGCTCTTACTTTCCTTGC
CCAGGGTGCCTCCCTGAAGCCTAGCGTGGTTGCTCGTGCAGCCATCAATGGTGTCATTGCCTGTGACACCACCACCT
TTCAACTCCTGGCGATGGATGGGACTCCCCTATCTTTCGCCGCTGCAGCGGTTGTGGTGCGCCAATTTCAATTGGCC
CACAACCGCTTTGTCAAGACTGGCTTGCGCCAGTTTAACAAGGAGTGCACGAGGCGCTGCAACGCCTTCAACCTTAA
GAAAGCTGCTCGCCAGAGCAGTGGACGTAGAGCCGCCTGCAAGCGGATTCTTGCGTCCTTGGATGCAGACCTGCCAT
GTAGCAGGTCCTCTCGCAGGGCCATAGTGACGGCCCATTATGCTGGAGTCGCAACTGCGGCTCGGAAGAGTGCTCTC
TTCCGCCAAATGCGCGCAGACAAGCGCGCATTTAGGGCTTTGGCAGCTGCCATGCAGCCCTTGGTTCTGGCTCCCCC
CACCTGTGCACGCATACCCTATGTGTGCCCACTTTCCTCTTCTGTGGAGGAAAGTGTTTCTCGACGCTCTCTTGAGC
GTTGTTCCAAGCGTGTTCCACGCGAACACGCTTCCCCCCGGGTTGCATTTGAAATGCAACCCTTTGCTGATCGTCCC
CGGCTTTTCTCCCGGGTGTTGCTCCTCCCTCTCTCAGAGATAACACGGGCGTTTTTCTCTCGTGTTTTTGTTCCTGG
GAGTGCCATGTATGATTGTGCTACTGATCTTGCACATGTTGTACTTGATTGGTGGTCCATCACCCCAATGGCACATT
ACTTGGTCATGCTTGACAATTTTCTTGGCACCCCGACTGAAGAAAGTGTGCGACAGGCTTCTACAAATCTTTTAGAG
GAAGTTGAAGCCATGCGTGCTCTTTGTAGAGACCACCGTGCGAACGGTGTGTTTGCTTGGGTGACAGAAACTGCTGA
TACGATTGGTAGCACCCTAAAGACTGTTGCTGCAGCTCCTTTCCATGGAGTTGGCGTTGCGCTTAAAACAGTTTTAA
CTCCTTGTGCTTCAGCTACTTTAGCGTGGGGGGAAAAATTTTTCCAGACCCTCAAATCCAAATTTTTTGAGTTCCTG
AAACCCTACATCCAACATGCTATTTATGCCAGTGCTGAAATTGAAAAATACTGGGCATTTATACATGGTTGGGCCAC
CAAGATGTGGAACAATGTTGGAGTAGAGTTGCAGGCGCTTGGTGATGCTGCATGGTGGGCCATTGGAATTACCATGG
TTTGTGGTATTGTTACCCTCGTTGAAAAGCTCTTAGTTTATCTTGGAGCTTTGAATGCGGGTGGTATATTGTGCTCT
CTAATGCTCACTGGTCTTCTAGGTGCTGCAGGTCTATTGGCGACTGGAAAATTTGCTGAGGCCAGTAGTACTCTTGT
TGGTGCCATGCGTTCTTTGATATTCACATTATTTGGATCATGGAAACCAACGGAGGCCAGTGATGGACTTACTTGCA
ATGCCAATGCTCTTGATTTCCCATTGAAAGTTTTGGAAACTGTAGGTACAGGGTTGATATCTGCTCCACTCGGTACA
CTACAGTATATTGGGAAATATGGGCAGGCTATGGACCAAATCCGAAAGGGCAAGGATGCCATCAAAGAGTTTGTTGG
ATTCTGCATGGATCGTGTAGCCGATGCTTGGGATTATATGACAGGTAGAAAGGATTCCTTTCTGCGTGAAATTGCGT
CCGCTGCAAAGGTTGATATTGTGTATTGGATCAAACAAACACAGAGTGTGCTGCTACAAGCTCAAACAATAGCGGTC
ACTGACATTGTCCTTTTGGACACAGTCACCCATTTGTTGTACAAAGGGCAAATACTGCAACTCACTCTTGCCAAGGC
TTCTCGCACAACATCTCTTGATTATGCACGTATTGTAAGTACGCTCATTGGTGAGCTTACAAAAATACGTGCAATAT
GTGCTCGTGCTGGAAGTTTTGATGGTCGTCGCCCAGAACCGTTTTGGTGCTATATTTACGGTAAGTCCCATTGCGGC
AAATCACTGTTTATGGAAGACGTTTCACGCGCTTTGCTTAAAGAGAATGGTCATGCTCCTAATGACATCTATGCGAA
GAATGCGCGTGATAGTTTTTGGTCCGGATATCTGCAGCACGCTTGCGTGCAGATAGATGATCTCTCGGCCTGTGTAA
CTCGACCCTCCCTTGAGTCTGAGTTTTTGCAGCTAGTGGGTTCTAAGATGTATTCCCTGAACATGGCTGCTGTTGAA
GACAAGGGTATGAGTTTTAATAGCTCGATCATTGTCACGACAGCAAATGTGTACACTGCTCCTACTAGCGCAGAAAT
TACTGATAAGGATGCATATGGAAATAGGCGGAATGTGGTAGTGCAGTGTCGAGCTGCCCCTGATGTTGAATTTGATC
CGCGCAATCCTTCCGCAAGTTGTGAAGCACGTTTAGTGCATCGCACTGATGAGAGCCCCCTAGGAAATGGGCAGTGG
```

AGAAACTGTTCCGCTGTGCTTGAGGATATCATTCACCACGCTGCCGTCCATCGCAACAAAGAGAATCTTCTTATGGA
GAATTATAGGGAGCGGTCGGACACGCAGCATCCAGTTTTTGTTGGGGCTAAAAGCTTTATCCATAGACTTGCAAAGG
AAAAGAACTTTGCTCACATAGTCTGTGATGATGTGCTCTATTACCACGACTCATACATTGATAGTACTCGTGTTTGT
GAGGGTACCATAAATATTGGCATGGAGGATGCATGTATACAGAGTGTGGTACAGTGGAGTGAATTAGTGGGTGGAGT
GAAGGACCTTGGTCTTCTCTACGCATTTGTACATGCTTTCACAGAGGGACCATGTCATGTTGATAGTGTTGAAGCTT
TGAATTCTGAAGCTACGAGTTGTCAGCGAGATTTTTTTCAATCGCTGTCGTTGCTGGAACGCATTTATATGCGACTT
GTACAAAAACAGCTCGATAGAATTCGGGCTAATCCAGATTTTTTTGTTTTCTGTAGATATTAAAACTAGAATTCTTC
AGAGTTTCCGTCATGGGTATGATGAAATGATTACACATGGCGGGAAGGTGTTGGCCATTTTTGCGGCCCTTCTTTTG
GTTTTGTTACTTTATTCTTCCTTCTTTGCTTTGTATCAGACATTTGTTGCTGGTACTAGTAGTGCCCTTGTGTCTGC
TGGTATGATAACCCAACTTAGCGCAAATGCTGGTTCGGTCTGCACTTCTGCGTCGAATCCTTCTGGAGCTGCCTCGT
ATGTATCGAGTAATATACCCATTCACCACAGGTGGCGTTCTAACTACTCTGAGAGATCATATGCGCTCAATTCCAAT
CTTGAGGATAAATATCTTCTTGATCTTCTCGTGTGGTTACAAATTCCAGGAGATTCGATAATCTCATGTATCCGTTT
CAAGGGAAGAAGTTTGCTGCTTACCAAACACCAGGCCCTAGCCATTCCTGAGGGGGCCCGTGTTTATTGCAATTACT
ATGGCCGTGGTCGCGCTGTACAAACAATCCCTCTGTCGTGGAGTTATAAGAAAGTGCGTGAATTTGCGGACACTGAA
GCGGTTCTTTTCCTTGATGCCCAGCTTTCGACAATGCCAGCAGGGCGGGAGCATTATTTCAACGTTCCTGTAGAACG
ATTGCCCACAGTATTTGACATGAATGGGGTCGTAATGAAACAGAAGCGTTACATGACGGACTCAGATGATAGCCTTG
CAGCTTTTACTCCCAATCAACCCGTTGTTAACACATGGGAGAATTCGCGTGCCAAACTCAACTGTGAGCGGCAAGGT
ATAAATACTTTTGCTTATGGAGGCAACTATAGAAATGAGCTACCACGCTCTATTTCTTCAAACTGTAACACCAGTCC
TGAGGATTGTGGTGCAATTATGACCATGATATTTGAGGGGAGAAGAATTGTCGTTGGCATGCATGTTGCCTCAGGGA
AGAACCCTCAAGGTCGCTATATGTCCACAGCATGTTTGCTCCCAGATTATCACGAGGACTTGTCGCTGAACTCCATG
CTTCAATATACACCATATGATGGTATTTGTAAGGAGGGTTACCGACAGATAGGAAATATTGAAAATATTGGTGCGCG
TCCATATACGTCAGGTAAAACTGCGTTTGTGGCCGTGCCCCAACATCTTTTATATAGTCCTCCGGTGTTGCAAGAGA
AGTTGCCTGGTAGTGCGCAATCTGTCACGATACAGGTTGAGGTGAAACAGCCAGCCATTTTGTCTAAGGATGATCCA
AGAATACCAGAGGGGACTTCCTATGACCCTTTGATTGACGGGATGGCCAAATTTTCACATCCTATGGCTGTACTTGA
TGAAAATGTGTGCGATGAAGTTGCACAGGATATAGTCGAATCGTGGCATGATTGCTTCCAAGACTTGCAAGACGTCT
CTGACGAGATTGCAATAAATGGTTCCACTGAGATGGACTATGAGCCCTTCAATCTTCAATCTTCGGAGGGATATCCT
TATGTGACTCAACGCAAACCAGGAGAAAGTGGCAAGATCCGTTTTTTTGAAATGGATCCCTATACTGGGCTCAAGAG
TCTCATTCCCAATACACTACCAGCTATGCGCTATGAAGCGTTGCAGCGGGATTGTTTCACTTCCGTTCCAGAAATGG
TGTGTATAGAGACGCCCAAAGATGAGTGCCTGCCTTTGCGTAAAATTTGTATCAAGCCCAAGACAAGGCTGTTCTCA
ATCTTGCCATTGGAGTTTAATCTCCTTCTCCGGAAAAAATTTCTCCACTTCTCCTCATCGCTCCAGATGCACCGTGA
CACCCTCCCCACCCAGGTAGGTGTGAATCCATATTCGAGGGAGTGGGGTGAGCTTTTGCAACGCTTGCGTGCTCAGA
GCAGCGTTGCTATAAATTGTGATTACGCCAGTTTTGATGGTCTGTTGACTGGTCAAATACTGGAGAAAATAGGTACC
ATGATCAACAAGATGTATATAGGGTCTGAAGCCTCTAAAACTCAGCGTTTAAATTTGCTCATGTCTATTGTGAATAG
AAAGAGTATATGTGGTGCGCGAGTTTATGAGGTGAGAGCGGGAATCCCTTCAGGATGTGCGCTGACAGTGCTGTTGA
ATTCCATATTTAATGAGTTTTTGATTCGTTTTGTATGGAGAACAACCATTATTGGTATTCCTCGGGAACGGTTTTCC
CAATATGTGACATTGTTGATCTATGGTGATGACAATCTCATAGCAGTACACCCCGATTATCTACCTCATTTTAATGG
GGAAATTATCCGGACGCGTCTCGCTGCCGTTAATGTCGTTATCACAGATGGTAGTGATAAGACTGCCGAGAAAATCG
AGGAAAAGCCATTGGTTCAGCTTGACTTTTTGAAACGTCGATTTAGGAAGTTGAATGATGGTACTGTCTATGCCCCA
CTTGATTTAGCATCGGTTTACACCAGTCTCCAAAATGTTACCATGGGAGCTGGCTCTATACATATGGCGTTGCAAAA
CAACGTCCATAATGCGCTCCTTGAGTTATATCTACACGGCAATGAGACATGGTTTAACCACTTAAGAGATTTCTATA
GGAAGTCTCATGCGTGGGTTAACCTTCCAAGTTGGCGTGAAGCTTTTGCATTTCACCAGGGACAAATTTCAGGAGTC
ACACCATGGACTCCCTATCAAATGTTTGACGTTCCTGTGGATGGTGAGCGCCTTAGAGCTATGATGGCTAATCAAGG
GGAAGCGACTTTTTCCACACACTTGGGCAGAGAAATATATATCTGCGGCCCAAAGTGGTGTGTTTCTGACCCAGAAC
ATCAATTCGTGGTGTCTACTACTCCCTTGAGGAGTGCAGACCGGGGCAGTGGTATTCACCGGGTTGTTGAATATCCC
TGTAACGGTGTGGGAAGACTGCCTACACAGGATTGGGTCGCCAAATTTAAGAGCTCCGCGCATAGAGTGACAGCTGA
AATCAGGAAAGCGCATGCAGGTGGAAAGGCGATATATTTTCGCGATGACCCACCGTATGTTGCTAACTGGTGTGCAG
CTATTGGGTTTGCACAGGGTTTGGGTTATGATTATAAATCTATGATTAACCTGTACCATGATGTATCTGTGCCAGGA
TCAGATGCTCTTTATTTTTATTTTGATCAACGGGCTCGGCGTGCTTTGCCTGAACCATATATCCCCCCCCATCTACG
CACGCGGGCTCGGTAGCCCAATAGTGGGGTTTGTAGATAAATAGCTAGTATCTTACTACTCGGACTCCCCTCTGCCG
TCATAGAGGGGTTTCAAGGGATGTCATCATCAGCGTTGGCTATGTCCGCGATGTGAATCATGGAGTAACGCACCATG
AGTCCCCCGTTAATTCGGTCCCGAGAGACTACACTTGGGTTGAAAATGATGGTAAACCATCCGTATGGGTCTATGTC
CATATGTTAAGAGTTGTCTTACTGTATATGGCTTCTACATAAGGTCGGTACCCCTGAAATAGGGATCCTGTTCGCTT
GCGTGGTTGTAGAAGTACTAAGTATGGTTGCAACATAAATATGACTTGGGAGACAAAGTCTCATCCACCTTGGAACC

GCACAGGGATATAACTCAAAGCGGAATGGTAGTGATCTACTTGCCCTATCAGTAGGGATGGGAACGCTCATTATTAG
CAAACCCTTACCCTGAACATTAGGTGTCTCAGCAATATGGGACAAAAGGCTAGGGAGAAAGATCCCATGGAAGTGCA
ATTCTTCTACTCGAGATGGGAGTCATGCCATCGTGTTATTTTATTTTCTTGCCTTGTATTTCAATTCTAAGAAAGTT
TCTGGATCTAAACCAGACCCAGGTGAGTGGAACCTTCTGCGTCCACTCGGTTTGGTATTTTACGATTAAAATACCCA
GGTCTACTGCTTCCGAGCCTGATTAAAATCTTAAAGCGGCCATCGTCCAGTACTTGCTGGCATGGCGACAAGAGTGA
TGATGCTTTGTGAATCATCAAATAGTTTCTTTGTTTCTATTTCTAGGAAATCCTTTTGGAGAGTGCCTAAGAGAATA
ACAAAGCGCATAGCACACGATAGAGTGCTATCTCTGTTCCAGGAGATTGAGCCGGAGATATAGTCGGTCGTTTCACG
ATCAAGGTATTTCTGGATGGGCCCGTTGCGAAGGGTTTTCGCTCAAGGTTCACATGACTGTAAATTGTAAGTGTACT
CAAGATGTAGCTAGTCTAGGCAACTAGTTACATTGGATGAGGTCCCGATTGATACGGGGGGATAATTAATCCCAGCT
TACGGTGTGTGCCGACTTGCATGGAAGTGTCCATGCCGCATTTTGCTTGTTTTTAGACATAAATAAGCACTCTTTAC
TGCGTATAATACGCTGGTGTCTCATTTTATACTAGCGTTGCAGCGCTAGGACTACCTAAGACAACCGTATTTTACGT
TTGCTTCATTTATTTTTGTTACTCCTGTTTAGCAGGTCGTGCCTTCAGTAAGCACACAAAAATATTTCCATTTTTA
GGTTTTGGTTAAGAGTCATTTTATTGAGCACTTTTCTTTTAGAGAATAGAGTCTGAA

>Cluster-12591.29271;orf1 len=1047 frame:3 start:3 end:3143
sp|Q8V5E0|POL1_BRAV RNA1 polyprotein OS=Blackcurrant reversion association virus OX=65743 PE=3 SV=1

FASSFSLSSFIDFTMVKIGSVTLAPKQLVLENKYIAACLSRPANFALTFLAQGASLKPSVVARAAINGVIACDTTTF
QLLAMDGTPLSFAAAAVVVRQFQLAHNRFVKTGLRQFNKECTRRCNAFNLKKAARQSSGRRAACKRILASLDADLPC
SRSSRRAIVTAHYAGVATAARKSALFRQMRADKRAFRALAAAMQPLVLAPPTCARIPYVCPLSSSVEESVSRRSLER
CSKRVPREHASPRVAFEMQPFADRPRLFSRVLLLPLSEITRAFFSRVFVPGSAMYDCATDLAHVVLDWWSITPMAHY
LVMLDNFLGTPTEESVRQASTNLLEEVEAMRALCRDHRANGVFAWVTETADTIGSTLKTVAAAPFHGVGVALKTVLT
PCASATLAWGEKFFQTLKSKFFEFLKPYIQHAIYASAEIEKYWAFIHGWATKMWNNVGVELQALGDAAWWAIGITMV
CGIVTLVEKLLVYLGALNAGGILCSLMLTGLLGAAGLLATGKFAEASSTLVGAMRSLIFTLFGSWKPTEASDGLTCN
ANALDFPLKVLETVGTGLISAPLGTLQYIGKYGQAMDQIRKGKDAIKEFVGFCMDRVADAWDYMTGRKDSFLREIAS
AAKVDIVYWIKQTQSVLLQAQTIAVTDIVLLDTVTHLLYKGQILQLTLAKASRTTSLDYARIVSTLIGELTKIRAIC
ARAGSFDGRRPEPFWCYIYGKSHCGKSLFMEDVSRALLKENGHAPNDIYAKNARDSFWSGYLQHACVQIDDLSACVT
RPSLESEFLQLVGSKMYSLNMAAVEDKGMSFNSSIIVTTANVYTAPTSAEITDKDAYGNRRNVVVQCRAAPDVEFDP
RNPSASCEARLVHRTDESPLGNGQWRNCSAVLEDIIHHAAVHRNKENLLMENYRERSDTQHPVFVGAKSFIHRLAKE
KNFAHIVCDDVLYYHDSYIDSTRVCEGTINIGMEDACIQSVVQWSELVGGVKDLGLLYAFVHAFTEGPCHVDSVEAL
NSEATSCQRDFFQSLSLLERIYMRLVQKQLDRIRANPDFFVFCRY*

RNA2 sequences in transcriptome of BRV-inoculated cv. Aldoniai

Underlined parts of sequences are translated to amino acids

>TRINITY_DN17768_c0_g1_i3 len=6369 path=[0:0-408 3:409-439 6:440-630 8:631-718 10:719-921 11:922-986 13:987-1032 14:1033-1062 15:1063-1245 17:1246-1281 18:1282-1371 20:1372-1409 22:1410-1629 23:1630-1667 25:1668-2595 26:2596-2600 27:2601-2739 29:2740-2774 30:2775-2905 32:2906-2945 33:2946-3747 35:3748-3788 36:3789-3924 38:3925-3977 40:3978-4374 41:4375-4406 43:4407-4815 44:4816-4850 45:4851-4899 47:4900-4940 49:4941-5057 50:5058-5067 51:5068-6368]

CTTTTCCCTGCTCTTTCCTTTTCCTTCTAGTTCCTTCTGTTCTGGTTTAATTGTTGTGGAGTAGGTTTTTCTTGGTC
TCCTTTGTCTTCCCTCTTTTGCCCTCCCCTCTTTTTCTTCTGATTTTGTTTGCTTTGAGATGAGCGAGTGTGGAGGT
GCCACACCCATGTCTGGGTGTGGTAGAATGTGTGCTCTCCGGAGCACCTGGTCAAAAAGGGCCTTCCTTACCGCCTG
TAAGGATGGTGCACTCACCAGTGATGGTAGGTGTCCGCAGTATGGTTGTGGGGCTCTTGTCTCCATTACCAAGGGTG
AACAGCAGCCCAAGAGAGCCGTCTCTGCAAAGGTGGCGAAATGCTTGTGCTGGGTCAAACCAGCACGTTGGTGTGAG
AAACACCAAGTGGGACCTGCAAGTCCCAATGGTTCTGCGGTCACCACAAGACCGAGATCTGCGCATGTAGCGCCGGC
CCCGCTTCCATATAGGAAGCAAACTTGTGACGTGGTTGTCACAGTTGGCCCTCTAGAGCTGGTTTATCCCGCTCTAG
TTTCTGAGGAGATACCAACTCCTGTCGCTGCCACCCCGACTAAGGTGGAAGAGGTTCCCATACCGGAGCTACCCTTG
TGGTTGGCTCCCACATGGATGGTGGAGCAGCCCTATGCTGCTACCCCAAAGGTCCCCCGCCTTACCCAACGGGAAGA
GTTTGCGCTACTTAAAAAGCGCCTTATCCGCAAGGGTAAGATCCTGCAGCGCCGTGCAACACACGCGCGCTGTGAAG
CTCGGGCTGCCTTAGTCCGTGTTCGTGCTGCCACCCAACGTAAGGTGGAGGAGGTCACCGCCCTTGTTGCCAAGGGC
AGGCGCATACTTGCCGCCCACCAGTTACTTCATGAACTGGAAGCGGTGGCCCCCCTTTCTCAAGCCCAAGAGCATCT
TGTGGCTTCTTCTTGTGCTGCAGCTGCAGCACGCCAGGAGGAATGCGCTAGCTTCCTTCGCCGCGCCAAGGCGTGGC
GGAAAAGCATAAGTGCAACTCCCCCTGTTGCTTTTGCCACAGCTGTGGCTTCTAAAGTGGCTTCGGTCACTTTGCCC
TGGGCCCATCTTGGGCTCTCTTTGCAGGGACTTTTGGCAGTCCCCACTCTTGATGGCACGTTGGGTGCCAAGCAATG
GTGTGCTAAAACCATTGCACAATGGGTCGTGACGCCCATTGTTTCTTGTGTCCGCTCTGCGCATGACAAAGTGCAGA
GCTGGCTACATAGCCAACCAGAAGTGGGTGTTATAAATACTAAGGTTCCTCTGGTGCAATCGGAGGTCAACTTGGAG
GTTTCATCACCTCTTTCTTTATCTGAAGAGATAGTGGATAAACCACGAGAAACTAGCCAGAGTGGAATCTGGCACCC
AGAGATGGGTGTAAGAAATATCTACGTTTTCCATGATGATTCATGGGAAACTAGTCTAGAAGAAGACGAAAAATTTA
CCTGTACTTATAATCGACGATGTGGTATTCCATATCTGCCGGTTGAAGGATGGGGCGCTGAAGAGCGTAAGAGTACT
ACCCCGGGATGGAATTTCTCTCTCCATAACAATGAAGAGTTCGAATTTCTTCCTAGTCCTGAAGAGGATTTTACTAA
GGAACTGGTTTTACCAGTCGCTCTTGAAGAGGAGGATAAATATTCCACAGCGAGTAGCAGTGGATTCTTCTCCTTGG
ACTCGACCACATCTAACATATGCGTGCAGTGTCCAGGGGTTTTGAGTGCTGATGCTGATGCCTACTTCTTTGATGGG
CCGGGCTATCGGTGCTCTTCGCGACCACGCGATTTTCGCTTACCAATTAGGAGAGGTTCTGACTATGAAAGCAGAGT
CAGAGACTCTATCAAGAGGAAAAGTGAAAATCCTTTGCAAGAGCGTTTTACCACTGTACTGAGGGAGAAGAGGAAAA
AGAAACACAAGAAAGAGTTTTATTCCTTCTCAGCATGCTTCGCTTTCAAGAGGAAGCAAATACAGTGGTCTCCCACA
GTCAACAAGATGTTTAATGATTGGGAGGACTATTGCGTTGCATCGGCATGGTTGCCTTTTGAAGTTGTTGTGACAGA
CGAAATTGAGAATGTCACACCTTTGTGTTCCAGTGGAGAGGATCACAATTGTAATAGTAGAGCGCCTTACCCGTTGG
CTCCAGTGTCGACGAAGTATTGTGACGATACTTGTTTTCATCCTAACGATGGGTGGACAACTGATGGGAATGGTAAA
CATTTCCGACTTAGTCCCTCGTTCGTTTTACCTGAAGTCCCCATACCAATAGTACAACGCACCACACACCGGTGTGC
GCAGTTTCTGCAAGATTGGGGTATTTATGATCTCACCTGTAATTCAAGCCTTCAAGCTGAAAATTCACAGAGTGAGA
TTCAGGAACGGATGGAAGATCGTGGTGAAGAGAAGCCGGTACCAAGCTTAGATATTCTTATTAAGAAACTTTCAAAG
AGGAATACTAAGGTAAAGGGTGCAGGCGAAAATCGCTATGCTGATCGTCATAGTCTAACTGAAAAGGCTATTTTCCA
CCAGCCCGGTGCGCTGAGCCGCATGCGCTCGGGTAAGGAAAAAACTATTGTGGCAGCGAATCACAACTCTGACCAGA
TTTCTGTACGCATGGCAGAATGCGGTAAACCCGTGTTCACACCATTGCCTCGGATGTCTGATGAAATGCTCAGAAGA
TTTCTCGAGAAAGGGTTGGGTTCTACTTCTACAGTGGCATTGGATATTGGCATACAAAGTCATATTCCACAGGGAAT
GCCTACTGTTGCGTTTGTGAATGTTATGGATACACGTATTGAGGATCCTTTATATTCTTCTTTGTGTGGTTCCTACA
TTGATCTTGGAAGGGATCGGGCAAAAACGCTATGTTTACCCCTTGTCAATTTCCCGATGAGCAAATTAGCAGAGGAT
GTTGATGATGTGTTAAATGGCTTGATGCTATGCACACATTTTCAAGATTCCACTAAATTTGGAGTTGGCAAACCTGC
TTTCCAATATGGCACGCTAGAATTCCAAGAATTTAAGCCATCTGCCTACTCGGATTTTTCCCGAGTTCGAGATAATT
GGGATGCGATCGCAAAACAACAGAATACACCAAATGATCGCATATTAGCAGGATTCTCTGTTCTTGGCGCTGTTTCA
CAAGCTTATAACCAAGCTCTGCCAGTTTTTAAATCTGTGGAGCTTGTGGCACCGCCCACGAGAAAGCCAGTAGTAGC
TACCTATCAAAATCCAACTACATTGGGTAGGAGTAGCACAACACGTAGTTTCCGCATGCCTACTTTGGATTTGCCGC

GGAGCACGGGGCGTGATGCTTCAATTCCTATAGTGCATCGGCGTAATAACAATGATGCACATGGATTTGATGAGGCT
ACACCAGCTCGTTTTTCAACATGTGACTCTGGATTGGTGGCTGACACCACGCTTGCATTTGCAAAGATGTATCAATG
CAAAAAAGATGCTAAAGCGGGGCATGTATTGGCCACTATTGATATCCAGAAATGTGTATTTGAAGACAATAGACGAG
TGGCTCTGGATTGGCTAGCACACGGTCTTGCTAGTTTTCAATATGATCTACAATTGACCGTAGGGTCGAACCCCTTT
GTTGGGGTAACCTTGGGGATTACAGTAGACGCTTTCGACCGGCTTCTGCCCCAGATTTCTGATGAGGTGATTGCGGT
GCCTTTGGCATTTCAACTGCCAACTTTTTTGTTCCCAATTTCAAAGAAGGGAACTTTCACTCAAACTATTGATTTTG
CGGCAATAGCTGGGTATAATTTTTTTCCGCATGTAGCAGCTTTTGGTCGGCCGAAAATTATCGTGTATATAGTGTCC
GATAATGATTTGCCAGCAAGTGACACATGGATGTGCCTTGTCGAGTTACACATGACTAGACTGGAAGCATCCACATT
AGCTTGTAGTCCCACCCTCATTTTGCCTCAAGCATTTGGGGGTGATTTACCTTTAGATTTGTGGCGTGGCCCATATA
CTTTTCCATTAGGGGGAGGTACCAAACGATTGTCTACTTCGTTGGATATTGGGCGGTCGACAACGACCGTTAGTGGG
TGGCGCACTGTTTCTTTTCCAGCTGCCTATGCTCTTTTCTTACAAGGGCATGGTGGTAGTCTTGTTGGTGAAGTAGT
GCATACTGGCTCAGCAGCTGTGTCTTGTGCTCTTCATCTTTGCATTTCTTTTGGTGGTGCTCCTCCTACTTTGGAGG
AAGCACTTGTTTTTCCTGGTTTTCGCTTACCTTCCGGTGAAGGCAAGTTTCATATCAAGGTACAGACGCCATATGGG
CGTTTAAGCACTTTAACTCCAGACTGTGCCTTATATGTTTACTTAGCTGGAGGACCCATAGCCGTTGCACCCATGTC
GGTGCCCTACCAATTCTGTATCCACTTGGAGAGGTTGGTAGACGATGGAGCCCCTCCTCGGACCATTGGTTTGATTC
GAGAGTTTAATTGGGCTACCATAAATAATTTCAAAAGTGATGACATTACGTTTGCTATTCCCGCGCGTTTGTCTGAT
CTTGTGTTGACTTGTGGAGATGTAACCATGAGCACTAACCCTCTTGCCCTACTGATTGGTAGTTGTGGCTTTTTCCG
AGGTAATCTTACGGTTGTCTTAGAATGGGCCACCTTTTTGAAGGCAGGAGATAAGGAGGGTACAGTACAGTTGACCA
CGTGTCGTGGCATGATCAATAATGTCAAGGGTGCGCGGAATGCCATTCAAAAGAAGGTTGTCAACTTGTCCCTCGTC
GGATCTATCTCACGTTACCTCAATGTTGGGGATTTCACCGGTTTTGCACAATCTGGTGGACAAGTGGGATATGATGA
AATTTTCCTCGAGTTCAGCACGAATAAAGCTAAACAGATCCGCTATCTGAATATAAATGTTGAACTCGATGAAAATT
TCGAGCTGTATGGTCGCACGGTTATACCTCTCAAAAATACAGCTCCCGCTCTCGCAAGCACATCTGCTAGCGCTCCC
AACACAAGTTGATAGCGATGTGTCCGTTTAGGAGATGAATAGCTAGTATCTTACTACTCGGATTCCCCTCTGCCGTC
ATAGAGGGGTTTCAAGGGATGTCATCATCAGCGTTGGCTATGTCCGCGATGTGAATCATGGAGTAACGCACCATGAG
TCCCCCGTTAATTCGGTCCCGAGAGACTACACTTGGGTTGAAAATGATGGTAAACCATCCGTATGGGTCTATGTCCA
TATGTTAAGAGTTGTCTTACTGTATATGGCTTCTACATAAGGTCGGTACCCCTGAAATAGGGATCCTGTTCGCTTGC
GTGGTTGTAGAAGTACTAAGTATGGTTGCAACATAAATATGACTTGGGAGACAAAGTCTCATCCACCTTGGAACCGC
ACAGGGATATAACTCAAAGCGGAATGGTAGTGATCTACTTGCCCTATCAGTAGGGATGGGAACGCTCATTATTAGCA
AACCCTTACCCTGAACATTAGGTGTCTCAGCAATATGGGACAAAAGGCTAGGGAGAAAGATCCCATGGAAGTGCAAT
TCTTCTACTCGAGATGGGAGTCATGCCATCGTGTTATTTTATTTTCTTGCCTTGTATTTCAATTCTAAGAAAGTTTC
TGGATCTAAACCAGACCCAGGTGAGTGGAACCTTCTGCGTCCACTCGGTTTGGTATTTTACGATTAAAATACCCAGG
TCTACTGCTTCCGAGCCTGATTAAAATCTTAAAGCGGCCATCGTCCAGTACTTGCTGGCATGGCGACAAGAGTGATG
ATGCTTTGTGAATCATCAAATAGTTTCTTTGTTTCTATTTCTAGGAAATCCTTTTGGAGAGTGCCTAAGAGAATAAC
AAAGCGCATAGCACACGATAGAGTGCTATCTCTGTTCCAGGAGATTGAGCCGGAGATATAGTCGGTCGTTTCACGAT
CAAGGTATTTCTGGATGGGCCCGTTGCGAAGGGTTTTCGCTCAAGGTTCACATGACTGTAAATTGTAAGTGTACTCA
AGATGTAGCTAGTCTAGGCAACTAGTTACATTGGATGAGGTCCCGATTGATACGGGGGGATAATTAATCCCAGCTTA
CGGTGTGTGCCGACTTGCATGGAAGTGTCCATGCCGCATTTTGCTTGTTTTTAGACATAAATAAGCACTCTTTACTG
CGTATAATACGCTGGTGTCTCATTTTATACTAGCGTTGCAGCGCTAGGACTACCTAAGACAACCGTATTTTACGTTT
GCTTCATTTATTTTTGTTACTCCTGTTTAGCAGGTCGTGCCTTCAGTAAGCACACAAAAATATTTTCCATTTTTAGG
TTTTGGTTAAGAGTCATTTTATTGAGCACTTTTCTTTTAGAGAATAGAGTCTGAA

>TRINITY_DN17768_c0_g1_i3 len=1626

VFLGLLCLPSFALPSFSSDFVCFEMSECGGATPMSGCGRMCALRSTWSKRAFLTACKDGALTSDGRCPQYGCGALVS
ITKGEQQPKRAVSAKVAKCLCWVKPARWCEKHQVGPASPNGSAVTTRPRSAHVAPAPLPYRKQTCDVVVTVGPLELV
YPALVSEEIPTPVAATPTKVEEVPIPELPLWLAPTWMVEQPYAATPKVPRLTQREEFALLKKRLIRKGKILQRRATH
ARCEARAALVRVRAATQRKVEEVTALVAKGRRILAAHQLLHELEAVAPLSQAQEHLVASSCAAAAARQEECASFLRR
AKAWRKSISATPPVAFATAVASKVASVTLPWAHLGLSLQGLLAVPTLDGTLGAKQWCAKTIAQWVVTPIVSCVRSAH
DKVQSWLHSQPEVGVINTKVPLVQSEVNLEVSSPLSLSEEIVDKPRETSQSGIWHPEMGVRNIYVFHDDSWETSLEE
DEKFTCTYNRRCGIPYLPVEGWGAEERKSTTPGWNFSLHNNEEFEFLPSPEEDFTKELVLPVALEEEDKYSTASSSG
FFSLDSTTSNICVQCPGVLSADADAYFFDGPGYRCSSRPRDFRLPIRRGSDYESRVRDSIKRKSENPLQERFTTVLR
EKRKKKHKKEFYSFSACFAFKRKQIQWSPTVNKMFNDWEDYCVASAWLPFEVVVTDEIENVTPLCSSGEDHNCNSRA
PYPLAPVSTKYCDDTCFHPNDGWTTDGNGKHFRLSPSFVLPEVPIPIVQRTTHRCAQFLQDWGIYDLTCNSSLQAEN

SQSEIQERMEDRGEEKPVPSLDILIKKLSKRNTKVKGAGENRYADRHSLTEKAIFHQPGALSRMRSGKEKTIVAANH
NSDQISVRMAECGKPVFTPLPRMSDEMLRRFLEKGLGSTSTVALDIGIQSHIPQGMPTVAFVNVMDTRIEDPLYSSL
CGSYIDLGRDRAKTLCLPLVNFPMSKLAEDVDDVLNGLMLCTHFQDSTKFGVGKPAFQYGTLEFQEFKPSAYSDFSR
VRDNWDAIAKQQNTPNDRILAGFSVLGAVSQAYNQALPVFKSVELVAPPTRKPVVATYQNPTTLGRSSTTRSFRMPT
LDLPRSTGRDASIPIVHRRNNNDAHGFDEATPARFSTCDSGLVADTTLAFAKMYQCKKDAKAGHVLATIDIQKCVFE
DNRRVALDWLAHGLASFQYDLQLTVGSNPFVGVTLGITVDAFDRLLPQISDEVIAVPLAFQLPTFLFPISKKGTFTQ
TIDFAAIAGYNFFPHVAAFGRPKIIVYIVSDNDLPASDTWMCLVELHMTRLEASTLACSPTLILPQAFGGDLPLDLW
RGPYTFPLGGGTKRLSTSLDIGRSTTTVSGWRTVSFPAAYALFLQGHGGSLVGEVVHTGSAAVSCALHLCISFGGAP
PTLEEALVFPGFRLPSGEGKFHIKVQTPYGRLSTLTPDCALYVYLAGGPIAVAPMSVPYQFCIHLERLVDDGAPPRT
IGLIREFNWATINNFKSDDITFAIPARLSDLVLTCGDVTMSTNPLALLIGSCGFFRGNLTVVLEWATFLKAGDKEGT
VQLTTCRGMINNVKGARNAIQKKVVNLSLVGSISRYLNVGDFTGFAQSGGQVGYDEIFLEFSTNKAKQIRYLNINVE
LDENFELYGRTVIPLKNTAPALASTSASAPNTS*

>TRINITY_DN17768_c0_g1_i4 len=6369 path=[0:0-408 3:409-439 6:440-630 8:631-718 10:719-921 11:922-986 13:987-1032 14:1033-1062 15:1063-1245 17:1246-1281 18:1282-1371 20:1372-1409 22:1410-1629 23:1630-1667 25:1668-2595 26:2596-2600 28:2601-2739 29:2740-2774 31:2775-2905 32:2906-2945 34:2946-3747 35:3748-3788 37:3789-3924 38:3925-3977 39:3978-4374 41:4375-4406 42:4407-4815 44:4816-4850 46:4851-4899 47:4900-4940 48:4941-5057 50:5058-5067 51:5068-6368]

CTTTTCCCTGCTCTTTCCTTTTCCTTCTAGTTCCTTCTGTTCTGGTTTAATTGTTGTGGAGTAGGTTTTTCTTGGTC
TCCTTTGTCTTCCCTCTTTTGCCCTCCCCTCTTTTTCTTCTGATTTTGTTTGCTTTGAGATGAGCGAGTGTGGAGGT
GCCACACCCATGTCTGGGTGTGGTAGAATGTGTGCTCTCCGGAGCACCTGGTCAAAAAGGGCCTTCCTTACCGCCTG
TAAGGATGGTGCACTCACCAGTGATGGTAGGTGTCCGCAGTATGGTTGTGGGGCTCTTGTCTCCATTACCAAGGGTG
AACAGCAGCCCAAGAGAGCCGTCTCTGCAAAGGTGGCGAAATGCTTGTGCTGGGTCAAACCAGCACGTTGGTGTGAG
AAACACCAAGTGGGACCTGCAAGTCCCAATGGTTCTGCGGTCACCACAAGACCGAGATCTGCGCATGTAGCGCCGGC
CCCGCTTCCATATAGGAAGCAAACTTGTGACGTGGTTGTCACAGTTGGCCCTCTAGAGCTGGTTTATCCCGCTCTAG
TTTCTGAGGAGATACCAACTCCTGTCGCTGCCACCCCGACTAAGGTGGAAGAGGTTCCCATACCGGAGCTACCCTTG
TGGTTGGCTCCCACATGGATGGTGGAGCAGCCCTATGCTGCTACCCCAAAGGTCCCCCGCCTTACCCAACGGGAAGA
GTTTGCGCTACTTAAAAAGCGCCTTATCCGCAAGGGTAAGATCCTGCAGCGCCGTGCAACACACGCGCGCTGTGAAG
CTCGGGCTGCCTTAGTCCGTGTTCGTGCTGCCACCCAACGTAAGGTGGAGGAGGTCACCGCCCTTGTTGCCAAGGGC
AGGCGCATACTTGCCGCCCACCAGTTACTTCATGAACTGGAAGCGGTGGCCCCCCTTTCTCAAGCCCAAGAGCATCT
TGTGGCTTCTTCTTGTGCTGCAGCTGCAGCACGCCAGGAGGAATGCGCTAGCTTCCTTCGCCGCGCCAAGGCGTGGC
GGAAAAGCATAAGTGCAACTCCCCCTGTTGCTTTTGCCACAGCTGTGGCTTCTAAAGTGGCTTCGGTCACTTTGCCC
TGGGCCCATCTTGGGCTCTCTTTGCAGGGACTTTTGGCAGTCCCCACTCTTGATGGCACGTTGGGTGCCAAGCAATG
GTGTGCTAAAACCATTGCACAATGGGTCGTGACGCCCATTGTTTCTTGTGTCCGCTCTGCGCATGACAAAGTGCAGA
GCTGGCTACATAGCCAACCAGAAGTGGGTGTTATAAATACTAAGGTTCCTCTGGTGCAATCGGAGGTCAACTTGGAG
GTTTCATCACCTCTTTCTTTATCTGAAGAGATAGTGGATAAACCACGAGAAACTAGCCAGAGTGGAATCTGGCACCC
AGAGATGGGTGTAAGAAATATCTACGTTTTCCATGATGATTCATGGGAAACTAGTCTAGAAGAAGACGAAAAATTTA
CCTGTACTTATAATCGACGATGTGGTATTCCATATCTGCCGGTTGAAGGATGGGGCGCTGAAGAGCGTAAGAGTACT
ACCCCGGGATGGAATTTCTCTCTCCATAACAATGAAGAGTTCGAATTTCTTCCTAGTCCTGAAGAGGATTTTACTAA
GGAACTGGTTTTACCAGTCGCTCTTGAAGAGGAGGATAAATATTCCACAGCGAGTAGCAGTGGATTCTTCTCCTTGG
ACTCGACCACATCTAACATATGCGTGCAGTGTCCAGGGGTTTTGAGTGCTGATGCTGATGCCTACTTCTTTGATGGG
CCGGGCTATCGGTGCTCTTCGCGACCACGCGATTTTCGCTTACCAATTAGGAGAGGTTCTGACTATGAAAGCAGAGT
CAGAGACTCTATCAAGAGGAAAAGTGAAAATCCTTTGCAAGAGCGTTTTACCACTGTACTGAGGGAGAAGAGGAAAA
AGAAACACAAGAAAGAGTTTTATTCCTTCTCAGCATGCTTCGCTTTCAAGAGGAAGCAAATACAGTGGTCTCCCACA
GTCAACAAGATGTTTAATGATTGGGAGGACTATTGCGTTGCATCGGCATGGTTGCCTTTTGAAGTTGTTGTGACAGA
CGAAATTGAGAATGTCACACCTTTGTGTTCCAGTGGAGAGGATCACAATTGTAATAGTAGAGCGCCTTACCCGTTGG
CTCCAGTGTCGACGAAGTATTGTGACGATACTTGTTTTCATCCTAACGATGGGTGGACAACTGATGGGAATGGTAAA
CATTTCCGACTTAGTCCCTCGTTCGTTTTACCTGAAGTCCCCATACCAATAGTACAACGCACCACACACCGGTGTGC
GCAGTTTCTGCAAGATTGGGGTATTTATGATCTCACCTGTAATTCAAGCCTTCAAGCTGAAAATTCACAGAGTGAGA
TTCAGGAACGGATGGAAGATCGTGGTGAAGAGAAGCCGGTACCAAGCTTAGATATTCTTATTAAGAAACTTTCAAAG
AGGAATACTAAGGTAAAGGGTGCAGGCGAAAATCGCTATGCTGATCGTCATAGTCTAACTGAAAAGGCTATTTTCCA

CCAGCCCGGTGCGCTGAGCCGCATGCGCTCGGGTAAGGAAAAACTATTGTGGCAGCGAACCATAACTCTGATCAGA
TTTCCGTACGCATGGCGGAATGTGGTAAGCCCATATTCACACCATTACCTCGGATGTCTGATGAAATGTTGAGAAAA
TTTTTGGAAAAAGGGTTAGGTTCCACTTCTACAGTGGCGTTGGACATTGGCATACAAAGTCATATTCCACAGGGAAT
GCCAACTGTTGCGTTTTTAAATGTTATGGACACACGCATTGAGGATCCCTTGTATTCTTCTTTGTGTGGTTCCTACA
TAGATCTTGGAAGGGATCGGGCTAAAACGCTTTGTTTACCTCTTGTCAACTTTCCATTGAGCAAATTAGCAGAGGAT
GTTGATGATGTGTTAAATGGTTTAATACTGTGTACACATTTTCAAGATTCTACTAAATTTGGAGTTGGTAAGCCTGC
TTTCCAATATGGCACTTTGGAGTTTCAGGAATTTAAGCCATCTGCCTATTCGGACTTTTCCCGTGTTCGAGATACTT
GGGACGCGATCGCTAAACAACAGAACACACCTAACGATCGTGTTTTAGCAGGATTTTCTGTTCTCGGTGCTGTGTCA
CAAGCGTATAATCAAGCTTTGCCGGACTTCAAGTCTATCGAACTTGTGGCACCACCTAAGAGGAAGCCTATTGTGGC
TACTTATCAAAATCCAACTACATTGGTGAGGAGCAATACCACCCGTAGCTTCCGCATGCCTACTATGGAATTGCCAC
GAAGTACGGGACGTGATACTCCCATTCCTATAGTGCATCGGCGTAACAACAATGATGCAAGTGGATTTGATGAAGCC
ACACCAGCTCGTTTCTCAACTTGTCAATCTGGACTTGTGGCTGACACTACGCTCGCTTTTGCAAAGATGTATCAATG
CAAGAAAGATGCCAAGGCGGGACATGTGTTGGCCACTGTTGATATTCAGACATGTGTCTTTGAGGACAATAGACGAG
TGGCTCTTGATTGGCTAGCACACGGTCTTGCTAGTTTCAAATATGATTTACAATTGACCGTGGGGTCGAACCCTTTC
GTAGGGGTAACTCTGGGGATTACAATAGATGCTTTCGACCGGCTTCTACCCCAAATTTCTGATGAGGTGATTGCAGT
GCCATTGGCGTTCCAGTTGCCTACTTTTTTGTTCCCAATCTCTAAGAAGGGGACTTTCACTCAAACTATTGATTTTG
CGGCAATAGCTGGGTACAATTTTTTTCCGCATGTAGCAGCATTTGGTCGGCCAAAAATTATCGTGTATATAGTGTCA
GATAATGATTTGCCAGCAAGTGATACGTGGATGTGCCTTCTCGAGTTACATATGACACGATTGGAATCATCCACTTT
AGCTTGTAGTCCCACCCTCATTTTGCCTCAAGCATTTGGGGGTGATTTACCCCTAGATTTGTGGCGTGGTCCATATA
CCTTTCCATTAGGGGGAGGTACCAAGCGCATATCTACTTCGTTAGATATTGGACGGTCAACAACGACTGTTAGTGGA
TGGCGCACTGTTTCTTTTCCTGCTGCCTATGCCTTGTTCTTACAGGGGCATGGTGGTAGTTTAGTTGGTGAGGTAGT
GCATACTGGCTCGGCTGCTGTGTCGTGTGCTCTTCACCTTTGTATATCTTTTGGTGGTGCTCCCCCTACTCTAGAGG
AAGCACTTGTTTTTCCTGGTTTTCGGCTACCTTCCGGTGAAGGCAAGTTTCATATTAAGGTACAGACGCCCTATGGG
CGCTTGAGCACTTTAACTCCTGACTGTGCCTTGTATGTCTACTTAGCTGGAGGACCTATTGCTGTTGCACCCATGTC
GGTGCCCTACCAATTCTGCATCCATCTGGAGAGATTGGTAGATGATGGAGCCCCTCCTCGAACCATTGGCTTGATTC
GAGAGTTTAACTGGGCTACCATAAATAATTTTAAGAGTGATGACATATCCTTTGCCATTCCGGCACGTTTGTCTGAT
CTTGTGCTCACTTGTGGTGATGTGACCATGAGTGTCAACCCTCTTGCCTTGTTAATTGGCAGTTGTGGTTTCTTCCG
GGGCAATCTTACAGTTGTTCTGGAATGGGCTACATTTCTAAAGGCGGGGGATAAGGAGGGCACAGTTCAATTGACTA
CGTGTCGTGGTATGATTAACAATGTTAAGGGTGTGCGTAGTGCCATCCAAAAGAAGGTTGTCAATCTATCACTTGTT
GGATCTATCTCACGATATCTCAACGTTGGAGATTTCACTGGCTTTGCACAATCTGGTGGACAAGTGGGATATGATGA
GATTTTTCTCGAGTTTAGCACGAATAAGGCTAAGCAAATTCGATATTTAAATATAAATGTTGAACTCGATGAAAATT
TCGAGCTGTATGGGCGTACTGTGATACCATTGAAAAATACAGCTCCCGTTCTCGCAAGCACATCAGCTAGTGCTCCC
AACGAAAGTTGATAGCGATGTGTACACCTTGGAGTTAAATAGCTAGTATCTTTCTACTCGGATTCCCCTCTGCCGTC
ATAGAGGGGTTTCAAGGGATGTCATCATCAGCGTTGGCTATGTCCGCGATGTGAATCATGGAGTAACGCACCATGAG
TCCCCCGTTAATTCGGTCCCGAGAGACTACACTTGGGTTGAAAATGATGGTAAACCATCCGTATGGGTCTATGTCCA
TATGTTAAGAGTTGTCTTACTGTATATGGCTTCTACATAAGGTCGGTACCCCTGAAATAGGGATCCTGTTCGCTTGC
GTGGTTGTAGAAGTACTAAGTATGGTTGCAACATAAATATGACTTGGGAGACAAAGTCTCATCCACCTTGGAACCGC
ACAGGGATATAACTCAAAGCGGAATGGTAGTGATCTACTTGCCCTATCAGTAGGGATGGGAACGCTCATTATTAGCA
AACCCTTACCCTGAACATTAGGTGTCTCAGCAATATGGGACAAAAGGCTAGGGAGAAAGATCCCATGGAAGTGCAAT
TCTTCTACTCGAGATGGGAGTCATGCCATCGTGTTATTTTATTTTCTTGCCTTGTATTTCAATTCTAAGAAAGTTTC
TGGATCTAAACCAGACCCAGGTGAGTGGAACCTTCTGCGTCCACTCGGTTTGGTATTTTACGATTAAAATACCCAGG
TCTACTGCTTCCGAGCCTGATTAAAATCTTAAAGCGGCCATCGTCCAGTACTTGCTGGCATGGCGACAAGAGTGATG
ATGCTTTGTGAATCATCAAATAGTTTCTTTGTTTCTATTTCTAGGAAATCCTTTTGGAGAGTGCCTAAGAGAATAAC
AAAGCGCATAGCACACGATAGAGTGCTATCTCTGTTCCAGGAGATTGAGCCGGAGATATAGTCGGTCGTTTCACGAT
CAAGGTATTTCTGGATGGCCCGTTGCGAAGGGTTTTCGCTCAAGGTTCACATGACTGTAAATTGTAAGTGTACTCA
AGATGTAGCTAGTCTAGGCAACTAGTTACATTGGATGAGGTCCCGATTGATACGGGGGGATAATTAATCCCAGCTTA
CGGTGTGTGCCGACTTGCATGGAAGTGTCCATGCCGCATTTTGCTTGTTTTTAGACATAAATAAGCACTCTTTACTG
CGTATAATACGCTGGTGTCTCATTTTATACTAGCGTTGCAGCGCTAGGACTACCTAAGACAACCGTATTTTACGTTT
GCTTCATTTATTTTTGTTACTCCTGTTTAGCAGGTCGTGCCTTCAGTAAGCACACAAAAATATTTTCCATTTTTAGG
TTTTGGTTAAGAGTCATTTTATTGAGCACTTTTCTTTTAGAGAATAGAGTCTGAA

>TRINITY_DN17768_c0_g1_i4

VFLGLLCLPSFALPSFSSDFVCFEMSECGGATPMSGCGRMCALRSTWSKRAFLTACKDGALTSDGRCPQYGCGALVS
ITKGEQQPKRAVSAKVAKCLCWVKPARWCEKHQVGPASPNGSAVTTRPRSAHVAPAPLPYRKQTCDVVVTVGPLELV
YPALVSEEIPTPVAATPTKVEEVPIPELPLWLAPTWMVEQPYAATPKVPRLTQREEFALLKKRLIRKGKILQRRATH
ARCEARAALVRVRAATQRKVEEVTALVAKGRRILAAHQLLHELEAVAPLSQAQEHLVASSCAAAAARQEECASFLRR
AKAWRKSISATPPVAFATAVASKVASVTLPWAHLGLSLQGLLAVPTLDGTLGAKQWCAKTIAQWVVTPIVSCVRSAH
DKVQSWLHSQPEVGVINTKVPLVQSEVNLEVSSPLSLSEEIVDKPRETSQSGIWHPEMGVRNIYVFHDDSWETSLEE
DEKFTCTYNRRCGIPYLPVEGWGAEERKSTTPGWNFSLHNNEEFEFLPSPEEDFTKELVLPVALEEEDKYSTASSSG
FFSLDSTTSNICVQCPGVLSADADAYFFDGPGYRCSSRPRDFRLPIRRGSDYESRVRDSIKRKSENPLQERFTTVLR
EKRKKKHKKEFYSFSACFAFKRKQIQWSPTVNKMFNDWEDYCVASAWLPFEVVVTDEIENVTPLCSSGEDHNCNSRA
PYPLAPVSTKYCDDTCFHPNDGWTTDGNGKHFRLSPSFVLPEVPIPIVQRTTHRCAQFLQDWGIYDLTCNSSLQAEN
SQSEIQERMEDRGEEKPVPSLDILIKKLSKRNTKVKGAGENRYADRHSLTEKAIFHQPGALSRMRSGKEKTIVAANH
NSDQISVRMAECGKPIFTPLPRMSDEMLRKFLEKGLGSTSTVALDIGIQSHIPQGMPTVAFLNVMDTRIEDPLYSSL
CGSYIDLGRDRAKTLCLPLVNFPLSKLAEDVDDVLNGLILCTHFQDSTKFGVGKPAFQYGTLEFQEFKPSAYSDFSR
VRDTWDAIAKQQNTPNDRVLAGFSVLGAVSQAYNQALPDFKSIELVAPPKRKPIVATYQNPTTLVRSNTTRSFRMPT
MELPRSTGRDTPIPIVHRRNNNDASGFDEATPARFSTCQSGLVADTTLAFAKMYQCKKDAKAGHVLATVDIQTCVFE
DNRRVALDWLAHGLASFKYDLQLTVGSNPFVGVTLGITIDAFDRLLPQISDEVIAVPLAFQLPTFLFPISKKGTFTQ
TIDFAAIAGYNFFPHVAAFGRPKIIVYIVSDNDLPASDTWMCLLELHMTRLESSTLACSPTLILPQAFGGDLPLDLW
RGPYTFPLGGGTKRISTSLDIGRSTTTVSGWRTVSFPAAYALFLQGHGGSLVGEVVHTGSAAVSCALHLCISFGGAP
PTLEEALVFPGFRLPSGEGKFHIKVQTPYGRLSTLTPDCALYVYLAGGPIAVAPMSVPYQFCIHLERLVDDGAPPRT
IGLIREFNWATINNFKSDDISFAIPARLSDLVLTCGDVTMSVNPLALLIGSCGFFRGNLTVVLEWATFLKAGDKEGT
VQLTTCRGMINNVKGVRSAIQKKVVNLSLVGSISRYLNVGDFTGFAQSGGQVGYDEIFLEFSTNKAKQIRYLNINVE
LDENFELYGRTVIPLKNTAPVLASTSASAPNES*

>TRINITY_DN17768_c0_g1_i2 len=6360 path=[1:0-402 3:403-433 5:434-624 8:625-712 9:713-915 11:916-980 12:981-1026 14:1027-1056 16:1057-1239 17:1240-1275 19:1276-1365 20:1366-1403 21:1404-1620 23:1621-1658 24:1659-2586 26:2587-2591 27:2592-2730 29:2731-2765 30:2766-2896 32:2897-2936 33:2937-3738 35:3739-3779 36:3780-3915 38:3916-3968 40:3969-4365 41:4366-4397 43:4398-4806 44:4807-4841 45:4842-4890 47:4891-4931 49:4932-5048 50:5049-5058 51:5059-6359]

ACTTTTCCCACCTCTTTCTTGTCATTTTTCTAATCTTCTTCCCTCCAGTTAGTATTGACCGATTGTTTGTCTTTTCC
TTTCATCTCTCTCTCTTTCTTCTGTTGTGGGGTTGCCTTTTGGTTTTCTCCCTATGAGTGATTGTGGTGGTACCACA
CCCATGCCTGGGTGTGGGAGAATGTGTGCTCTCAAGAGCATCTGGTCCCGAAGGGCCTTCCTTGCTGCCTGCAAGGA
TGGCGCACTTACCTCTGATGGTAGGTGTCCACAATATGGTTGTGGGGTTCTTGTCCCCATTACCAAGGGTGTACAGC
AGCCCAAGAAAACCGCTTCTGCAAAGGCGGTAAAATGCTTGTGCTGGGTTCAACCAGCACGCTGGTGCGAAAAGCAT
CAAAAGGGGCCTGCAAGCCCCAATGGTTCTGCGGTCACCACAAGACCGAAGTCTGCGCATGTAGCGCCAGCCCCGCT
TCCTTACAAGAAGCAGATTTGTGACGTGGTGGTCACAGTTGGGCCTCTAGAGTTGGTTTATCCAGCTCTAGTTTCTG
GGGAGACACCAGCTCCCATCGCTGCCACCCCGATTGAGGTGGAAGAGGTTCCCATACCGGAGCTGCCCTTGTGGTTA
GCTCCGGCGTGGATGGTGGAGCAGCCCTATGCTGCTACCCCAAAGGTCCCCCGCCTTACCCAACGGGAAGAGTTTGC
GCTACTTAAAAAGCGCCTTACCCGTAAGGGTAAGCTTATACAACGCCGTGCCACGCACGCGCGTTTTGTGGCCCGGG
CTACATTGGCCCGTGTCCGTGCTGCCACCCAACGTAAGGTGGACGAGATTACCGCCCTTGTTGTCAAGGGTAGGCGG
ATTTTGGCCGCCCACCAACTCCTTCGTGAGTTGGAAGAGGTAACCCCTCTCTCTCAAGCCCAAGAACAACTTGTGGC
TTCTTCTTGTGCTGCAGCTGCAGCACGCCAGGAGGAATGCGCTAGCTTCCTTCGCCGTGCCAAAGCATGGCGGAGGA
GCATTAGTGCAACTCCTCCTGTTGCCTTTGCCACAGCTGTGGCTTCTAAAGTGGCTACGGCCACCATGCCTTGGGCT
CATCTTGGGCTCTCTCTTGGGGGACTTTTGGCAGTCCCAACTTTGGATGGCACTCTGGGTGCCAAGCAATGGAATGC
TAAAACCATTGCTACATGGGTTGTAAAACCTATTGTTTCCTGTATCCGTTCTGCACATAACAAAGTGCGGGACTGGT
TACGTAGTCAACCAGAAGTGGGTGTTATAAATACTAAGGTTCCTTCAGTGCAACTGGAGGTCTGCTCGGAGGTTTCA
TTACCTCCCTTGTTATCTGAAGAGATAGTGGATAAACCACAAGAAACTAGCCAGAGCGGAATCTGGCACCCAGAGAT
GGGTGTAAGAAATATCTATGTTTTTCACGATGATTCGTGGGAAACTAGTCCAGAAGAGGACGATAAATTTACCTATA
CTTTTAGTCGACAATGTGGTATTCCATATTTGCCGGTTGAGGGTTGGGGCGCTGAAGAGCGTAAGAGTACTATTCTT
GGTTGGGATTTCCCTCTCTATGATGGAGAGTTTGAATTCTTTCCCAGTCCTGAAGAGGATAATACTAAGGAACTGGT
TTTGCCAGTCGCTCTTGAAGAGGAGGATAAATATTCCACAGCAAGTAGCAGTGGATTCTTCTCCCTGAACTCCATTG

TGTCTGATATATATGCAGATTGTTCAGGGGTTGTGAGCACCAGGACTTGCGCCCGTTTTCGTGACGGAACAGGTTTT
CGGGGCTTTTCGCGACCACGTGATTTTCGCCCACCTATAAAGAGGAGCTCAGACTATGAAAGTAAAGTCAGAGCTTC
AATTCAAAGGAAGGTTGAAAATCCCTTGCAAGAACGCTTTACCACTGTGGTAAGAGAGAAGCGGAAGAAGAAGCAAA
AGAAAGAATTTTATTCCTTCTCAGCTTATTTCGCTTTTAAAAGGAAGCAGATACAGTGGCCTCCCTCAGCTAGTAAA
ATGTTTAATGAATGGGAGGATTATTGCGTTGCAGCGGCGTGGTTGCCTTTTGAAGTTGTAGTGACAGACGAAATTGA
GAATGTCACTCCTTTGTGCTCCGGTGGAGGAGATCACAATTGTAATAGTAGACCACCTCGCCCGTTGGCTCCATTGT
CAACGGTTTTCTGTGATAGTTCGTGTTTTCATCCTAATGACGGGTGGACGACTGGAGAAAATGGGAAACATTTTCGA
CTTGGTCCCTCGTTCGTTTTACCCGAACCCCCCATACCAATAGTGCACCGCGTTGCACGCCAATGCGCGCAGCTCTG
TCAAGATTGGGGTATTGGTGATCTCACTTGCAATTCAGGCTACCAAGCTGACAATTTGCAGGGAGAGATTCAAGAAC
GGATGGAAGATCGCGGTGAAGAAAAACCAGTGCCGAGTCTAGACACTCTTATTAAGAAACTTTCGAAGAGGAGTGCT
AAGGTTAAAGGTGCAGGTGAAAATCGATACGCCGATCGTCACAGTCTAACCGAGAAGGCTATTTTCCACCAACCTGG
AGCGTTGAGCCGCATGCGTTCAGGTAAAGAAAAAACTATTGTGGCGGCGAATCACAACTCTGACCAGATTTCTGTAC
GCATGGCAGAATGCGGTAAACCCGTGTTCACACCATTGCCTCGGATGTCTGATGAAATGCTCAGAAGATTTCTCGAG
AAAGGGTTGGGTTCTACTTCTACAGTGGCATTGGATATTGGCATACAAAGTCATATTCCACAGGGAATGCCTACTGT
TGCGTTTGTGAATGTTATGGATACACGTATTGAGGATCCTTTATATTCTTCTTTGTGTGGTTCCTACATTGATCTTG
GAAGGGATCGGGCAAAAACGCTATGTTTACCCCTTGTCAATTTCCCGATGAGCAAATTAGCAGAGGATGTTGATGAT
GTGTTAAATGGCTTGATGCTATGCACACATTTTCAAGATTCCACTAAATTTGGAGTTGGCAAACCTGCTTTCCAATA
TGGCACGCTAGAATTCCAAGAATTTAAGCCATCTGCCTACTCGGATTTTTCCCGAGTTCGAGATAATTGGGATGCGA
TCGCAAAACAACAGAATACACCAAATGATCGCATATTAGCAGGATTCTCTGTTCTTGGCGCTGTTTCACAAGCTTAT
AACCAAGCTCTGCCAGTTTTTAAATCTGTGGAGCTTGTGGCACCGCCCACGAGAAAGCCAGTAGTAGCTACCTATCA
AAATCCAACTACATTGGGTAGGAGTAGCACAACACGTAGTTTCCGCATGCCTACTTTGGATTTGCCGCGGAGCACGG
GGCGTGATGCTTCAATTCCTATAGTGCATCGGCGTAATAACAATGATGCACATGGATTTGATGAGGCTACACCAGCT
CGTTTTTCAACATGTGACTCTGGATTGGTGGCTGACACCACGCTTGCATTTGCAAAGATGTATCAATGCAAAAAAGA
TGCTAAAGCGGGGCATGTATTGGCCACTATTGATATCCAGAAATGTGTATTTGAAGACAATAGACGAGTGGCTCTGG
ATTGGCTAGCACACGGTCTTGCTAGTTTTCAATATGATCTACAATTGACCGTAGGGTCGAACCCCTTTGTTGGGGTA
ACCTTGGGGATTACAGTAGACGCTTTCGACCGGCTTCTGCCCCAGATTTCTGATGAGGTGATTGCGGTGCCTTTGGC
ATTTCAACTGCCAACTTTTTTGTTCCCAATTTCAAAGAAGGGAACTTTCACTCAAACTATTGATTTTGCGGCAATAG
CTGGGTATAATTTTTTTCCGCATGTAGCAGCTTTTGGTCGGCCGAAAATTATCGTGTATATAGTGTCCGATAATGAT
TTGCCAGCAAGTGACACATGGATGTGCCTTGTCGAGTTACACATGACTAGACTGGAAGCATCCACATTAGCTTGTAG
TCCCACCCTCATTTTGCCTCAAGCATTTGGGGGTGATTTACCTTTAGATTTGTGGCGTGGCCCATATACTTTTCCAT
TAGGGGGAGGTACCAAACGATTGTCTACTTCGTTGGATATTGGGCGGTCGACAACGACCGTTAGTGGGTGGCGCACT
GTTTCTTTTCCAGCTGCCTATGCTCTTTTCTTACAAGGGCATGGTGGTAGTCTTGTTGGTGAAGTAGTGCATACTGG
CTCAGCAGCTGTGTCTTGTGCTCTTCATCTTTGCATTTCTTTTGGTGGTGCTCCTCCTACTTTGGAGGAAGCACTTG
TTTTTCCTGGTTTTCGCTTACCTTCCGGTGAAGGCAAGTTTCATATCAAGGTACAGACGCCATATGGGCGTTTAAGC
ACTTTAACTCCAGACTGTGCCTTATATGTTTACTTAGCTGGAGGACCCATAGCCGTTGCACCCATGTCGGTGCCCCTA
CCAATTCTGTATCCACTTGGAGAGGTTGGTAGACGATGGAGCCCCTCCTCGGACCATTGGTTTGATTCGAGAGTTTA
ATTGGGCTACCATAAATAATTTCAAAAGTGATGACATTACGTTTGCTATTCCCGCGCGTTTGTCTGATCTTGTGTTG
ACTTGTGGAGATGTAACCATGAGCACTAACCCTCTTGCCCTACTGATTGGTAGTTGTGGCTTTTTCCGAGGTAATCT
TACGGTTGTCTTAGAATGGGCCACCTTTTTGAAGGCAGGAGATAAGGAGGGTACAGTACAGTTGACCACGTGTCGTG
GCATGATCAATAATGTCAAGGGTGCGCGGAATGCCATTCAAAAGAAGGTTGTCAACTTGTCCCTCGTCGGATCTATC
TCACGTTACCTCAATGTTGGGGATTTCACCGGTTTTGCACAATCTGGTGGACAAGTGGGATATGATGAAATTTTCCT
CGAGTTCAGCACGAATAAAGCTAAACAGATCCGCTATCTGAATATAAATGTTGAACTCGATGAAAATTTCGAGCTGT
ATGGTCGCACGGTTATACCTCTCAAAAATACAGCTCCCGCTCTCGCAAGCACATCTGCTAGCGCTCCCAACACAAGT
TGATAGCGATGTGTCCGTTTAGGAGATGAATAGCTAGTATCTTACTACTCGGATTCCCCTCTGCCGTCATAGAGGGG
TTTCAAGGGATGTCATCATCAGCGTTGGCTATGTCCGCGATGTGAATCATGGAGTAACGCACCATGAGTCCCCCGTT
AATTCGGTCCCGAGAGACTACACTTGGGTTGAAAATGATGGTAAACCATCCGTATGGGTCTATGTCCATATGTTAAG
AGTTGTCTTACTGTATATGGCTTCTACATAAGGTCGGTACCCCTGAAATAGGGATCCTGTTCGCTTGCGTGGTTGTA
GAAGTACTAAGTATGGTTGCAACATAAATATGACTTGGGAGACAAAGTCTCATCCACCTTGGAACCGCACAGGGATA
TAACTCAAAGCGGAATGGTAGTGATCTACTTGCCCTATCAGTAGGGATGGGAACGCTCATTATTAGCAAACCCTTAC
CCTGAACATTAGGTGTCTCAGCAATATGGGACAAAAGGCTAGGGAGAAAGATCCCATGGAAGTGCAATTCTTCTACT
CGAGATGGGAGTCATGCCATCGTGTTATTTTATTTTCTTGCCTTGTATTTCAATTCTAAGAAAGTTTCTGGATCTAA
ACCAGACCCAGGTGAGTGGAACCTTCTGCGTCCACTCGGTTTGGTATTTTACGATTAAAATACCCAGGTCTACTGCT
TCCGAGCCTGATTAAAATCTTAAAGCGGCCATCGTCCAGTACTTGCTGGCATGGCGACAAGAGTGATGATGCTTTGT

GAATCATCAAATAGTTTCTTTGTTTCTATTTCTAGGAAATCCTTTTGGAGAGTGCCTAAGAGAATAACAAAGCGCAT
AGCACACGATAGAGTGCTATCTCTGTTCCAGGAGATTGAGCCGGAGATATAGTCGGTCGTTTCACGATCAAGGTATT
TCTGGATGGGCCCGTTGCGAAGGGTTTTCGCTCAAGGTTCACATGACTGTAAATTGTAAGTGTACTCAAGATGTAGC
TAGTCTAGGCAACTAGTTACATTGGATGAGGTCCCGATTGATACGGGGGGATAATTAATCCCAGCTTACGGTGTGTG
CCGACTTGCATGGAAGTGTCCATGCCGCATTTTGCTTGTTTTTAGACATAAATAAGCACTCTTTACTGCGTATAATA
CGCTGGTGTCTCATTTTATACTAGCGTTGCAGCGCTAGGACTACCTAAGACAACCGTATTTTACGTTTGCTTCATTT
ATTTTTGTTACTCCTGTTTAGCAGGTCGTGCCTTCAGTAAGCACACAAAAATATTTTCCATTTTTAGGTTTTGGTTA
AGAGTCATTTTATTGAGCACTTTTCTTTTAGAGAATAGAGTCTGAA

>TRINITY_DN17768_c0_g1_i2

PIVCLFLSSLSLSSVVGLPFGFLPMSDCGGTTPMPGCGRMCALKSIWSRRAFLAACKDGALTSDGRCPQYGCGVLVP
ITKGVQQPKKTASAKAVKCLCWVQPARWCEKHQKGPASPNGSAVTTRPKSAHVAPAPLPYKKQICDVVVTVGPLELV
YPALVSGETPAPIAATPIEVEEVPIPELPLWLAPAWMVEQPYAATPKVPRLTQREEFALLKKRLTRKGKLIQRRATH
ARFVARATLARVRAATQRKVDEITALVVKGRRILAAHQLLRELEEVTPLSQAQEQLVASSCAAAAARQEECASFLRR
AKAWRRSISATPPVAFATAVASKVATATMPWAHLGLSLGGLLAVPTLDGTLGAKQWNAKTIATWVVKPIVSCIRSAH
NKVRDWLRSQPEVGVINTKVPSVQLEVCSEVSLPPLLSEEIVDKPQETSQSGIWHPEMGVRNIYVFHDDSWETSPEE
DDKFTYTFSRQCGIPYLPVEGWGAEERKSTILGWDFPLYDGEFEFFPSPEEDNTKELVLPVALEEEDKYSTASSSGF
FSLNSIVSDIYADCSGVVSTRTCARFRDGTGFRGFSRPRDFRPPIKRSSDYESKVRASIQRKVENPLQERFTTVVRE
KRKKKQKKEFYSFSAYFAFKRKQIQWPPSASKMFNEWEDYCVAAAWLPFEVVVTDEIENVTPLCSGGGDHNCNSRPP
RPLAPLSTVFCDSSCFHPNDGWTTGENGKHFRLGPSFVLPEPPIPIVHRVARQCAQLCQDWGIGDLTCNSGYQADNL
QGEIQERMEDRGEEKPVPSLDTLIKKLSKRSAKVKGAGENRYADRHSLTEKAIFHQPGALSRMRSGKEKTIVAANHN
SDQISVRMAECGKPVFTPLPRMSDEMLRRFLEKGLGSTSTVALDIGIQSHIPQGMPTVAFVNVMDTRIEDPLYSSLC
GSYIDLGRDRAKTLCLPLVNFPMSKLAEDVDDVLNGLMLCTHFQDSTKFGVGKPAFQYGTLEFQEFKPSAYSDFSRV
RDNWDAIAKQQNTPNDRILAGFSVLGAVSQAYNQALPVFKSVELVAPPTRKPVVATYQNPTTLGRSSTTRSFRMPTL
DLPRSTGRDASIPIVHRRNNNDAHGFDEATPARFSTCDSGLVADTTLAFAKMYQCKKDAKAGHVLATIDIQKCVFED
NRRVALDWLAHGLASFQYDLQLTVGSNPFVGVTLGITVDAFDRLLPQISDEVIAVPLAFQLPTFLFPISKKGTFTQT
IDFAAIAGYNFFPHVAAFGRPKIIVYIVSDNDLPASDTWMCLVELHMTRLEASTLACSPTLILPQAFGGDLPLDLWR
GPYTFPLGGGTKRLSTSLDIGRSTTTVSGWRTVSFPAAYALFLQGHGGSLVGEVVHTGSAAVSCALHLCISFGGAPP
TLEEALVFPGFRLPSGEGKFHIKVQTPYGRLSTLTPDCALYVYLAGGPIAVAPMSVPYQFCIHLERLVDDGAPPRTI
GLIREFNWATINNFKSDDITFAIPARLSDLVLTCGDVTMSTNPLALLIGSCGFFRGNLTVVLEWATFLKAGDKEGTV
QLTTCRGMINNVKGARNAIQKKVVNLSLVGSISRYLNVGDFTGFAQSGGQVGYDEIFLEFSTNKAKQIRYLNINVEL
DENFELYGRTVIPLKNTAPALASTSASAPNTS*
