# Supplementary figures and images for: De Novo Transcriptome Analysis of R. nigrum cv. Aldoniai in Response to Blackcurrant Reversion Virus Infection
Source: Int J Mol Sci. 2022 Aug 24;23(17):9560. doi: 10.3390/ijms23179560 (PMC9455767; doi:10.3390/ijms23179560)

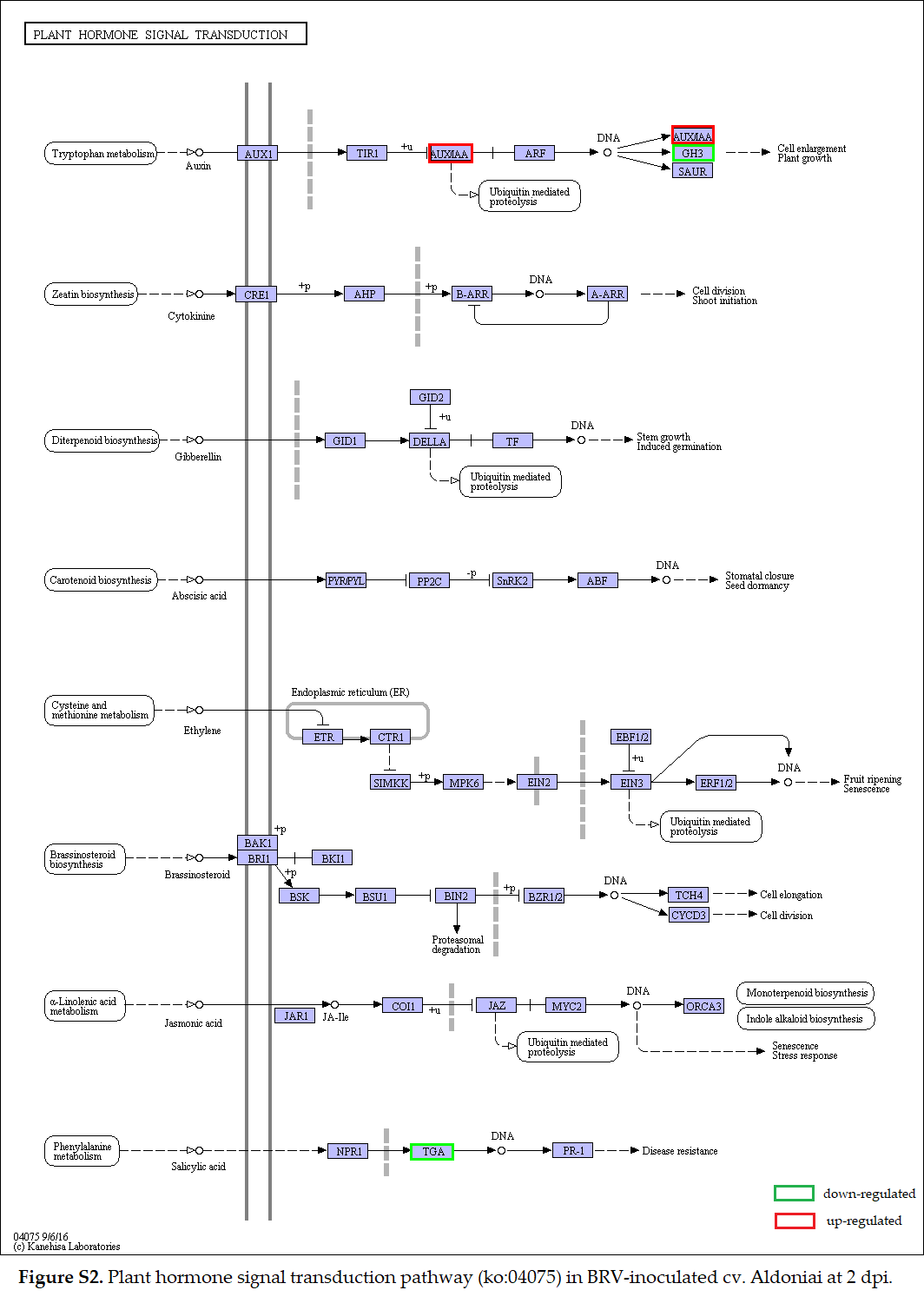

Supplement: Supplementary file 1 [file ijms-23-09560-s001.zip › Supplements/Figure S2.png]

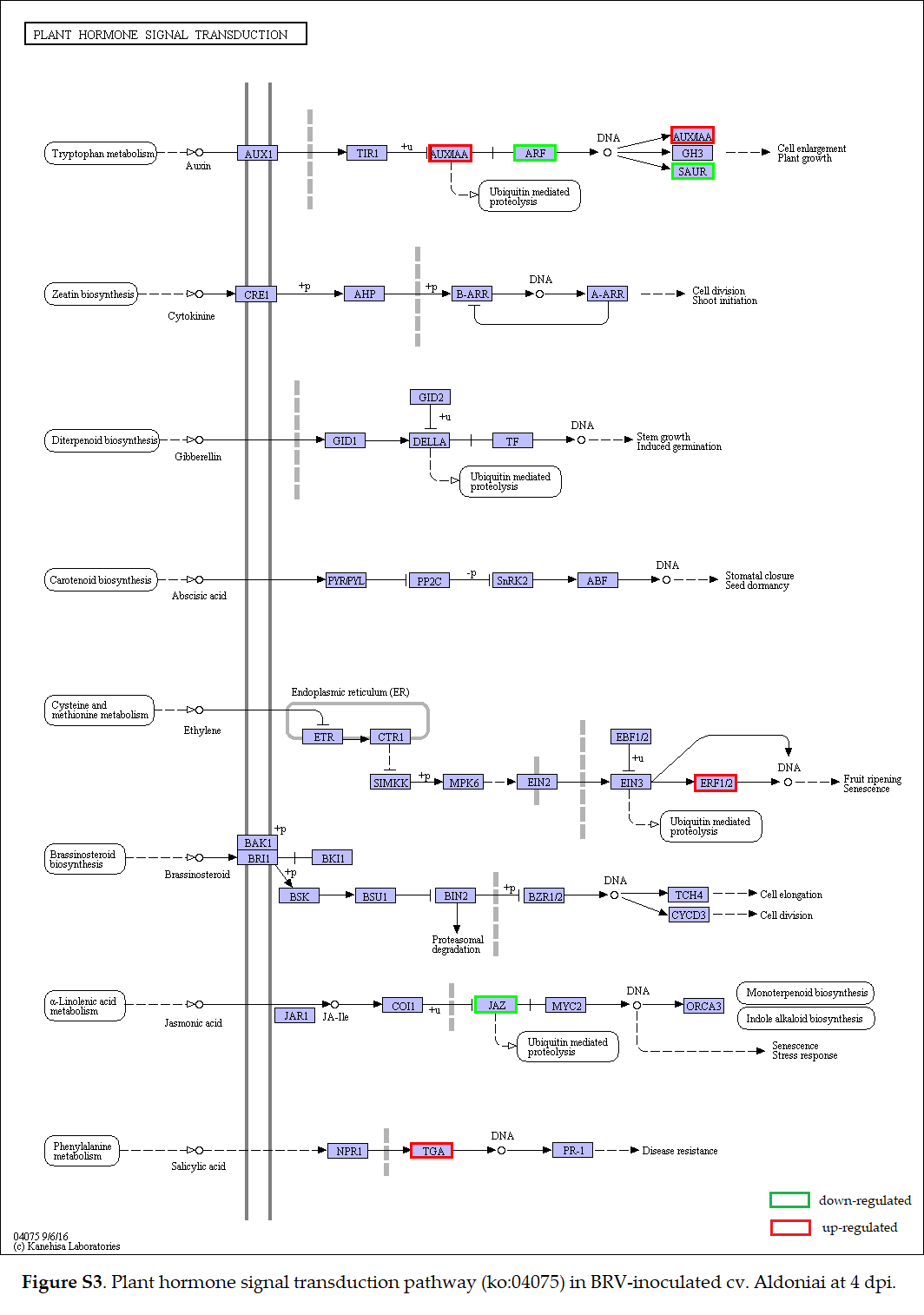

Supplement: Supplementary file 1 [file ijms-23-09560-s001.zip › Supplements/Figure S3.png]

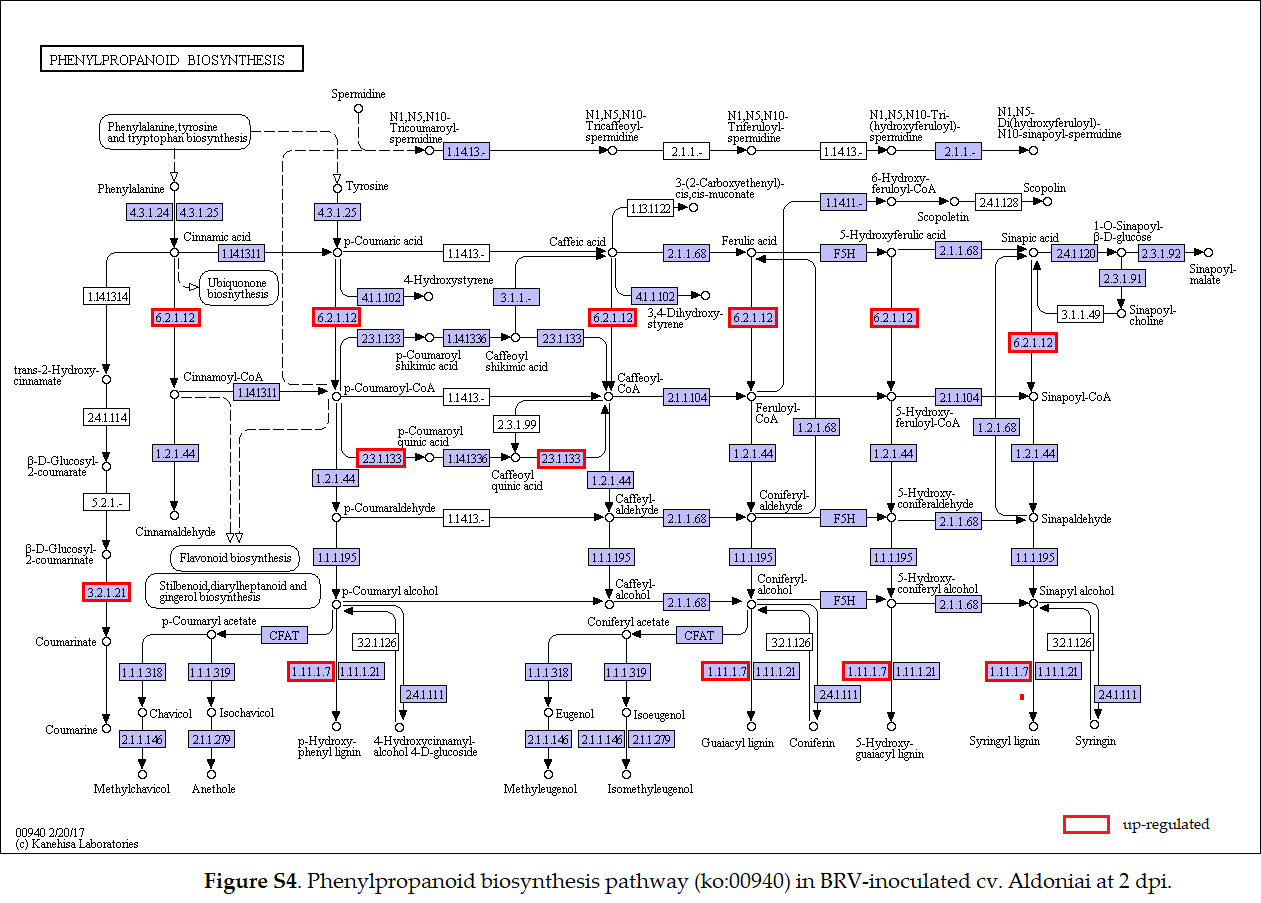

Supplement: Supplementary file 1 [file ijms-23-09560-s001.zip › Supplements/Figure S4.png]

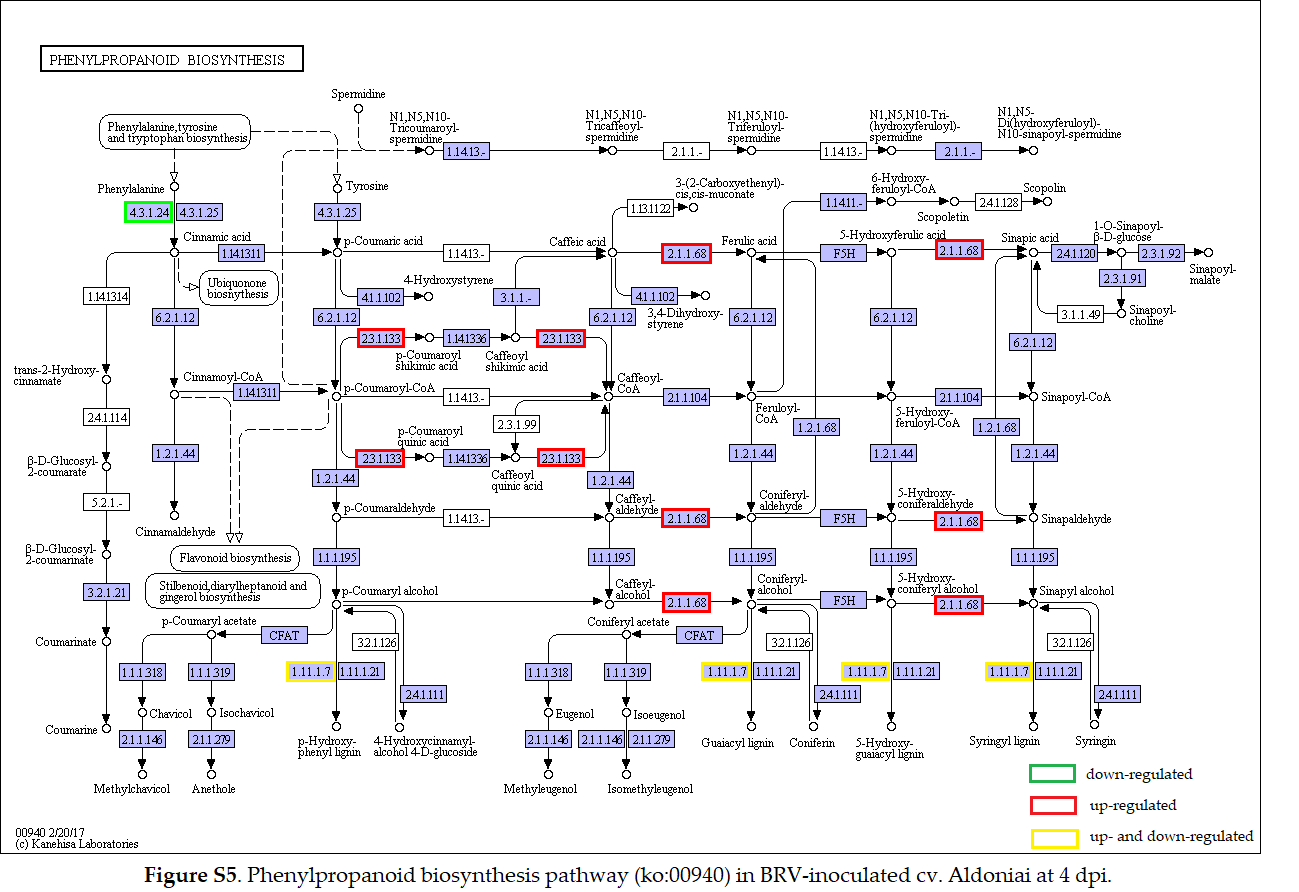

Supplement: Supplementary file 1 [file ijms-23-09560-s001.zip › Supplements/Figure S5.png]

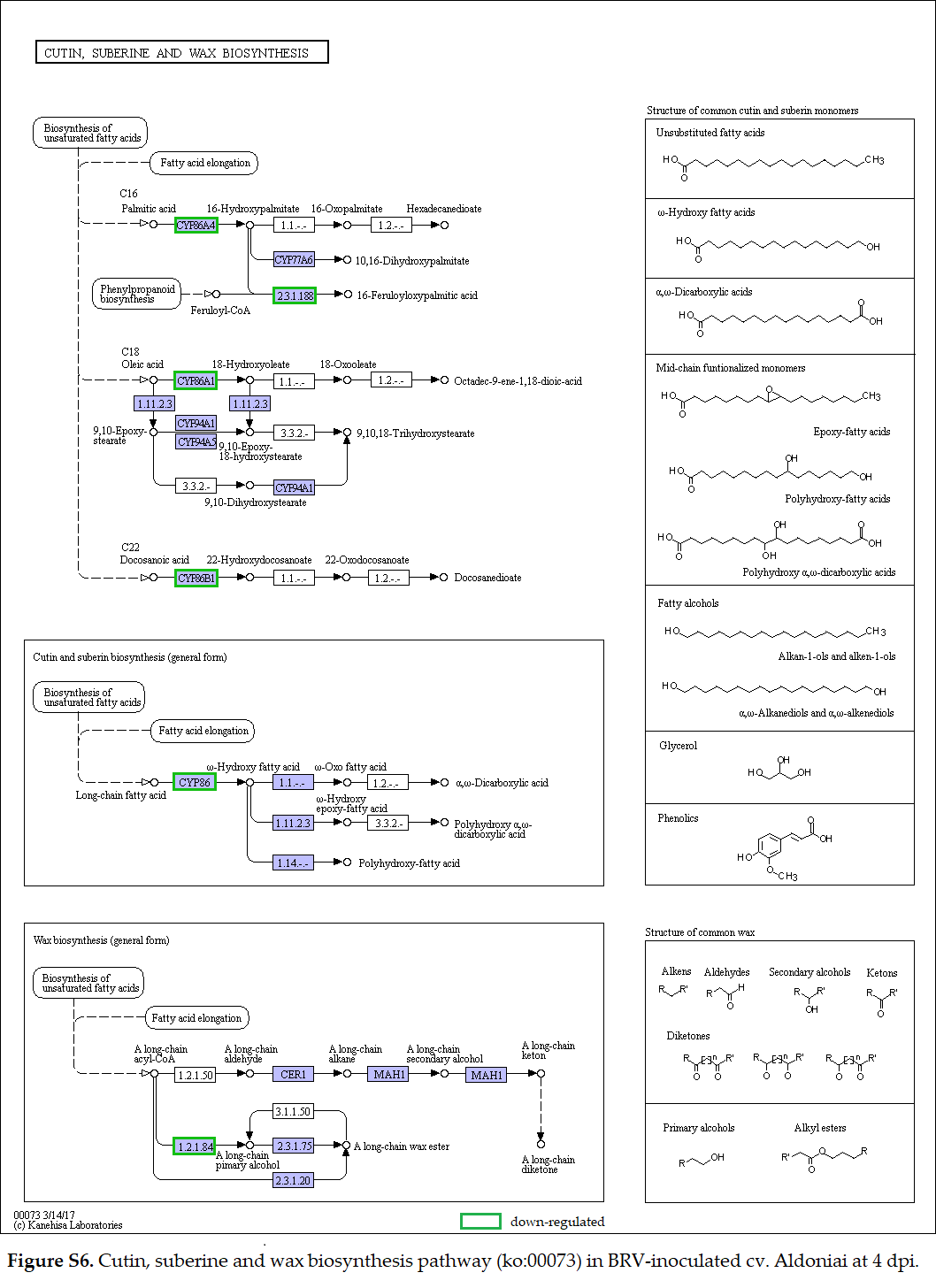

Supplement: Supplementary file 1 [file ijms-23-09560-s001.zip › Supplements/Figure S6.png]
